# A novel tumour suppressor protein encoded by circMAPK14 inhibits progression and metastasis of colorectal cancer by competitively binding to MKK6

**DOI:** 10.1002/ctm2.613

**Published:** 2021-10-14

**Authors:** Lu Wang, Jiahui Zhou, Chuan Zhang, Ranran Chen, Qingyang Sun, Peng Yang, Chaofan Peng, Yuqian Tan, Chi Jin, Tuo Wang, Jiangzhou Ji, Yueming Sun

**Affiliations:** ^1^ Division of Colorectal Surgery Department of General Surgery The First Affiliated Hospital of Nanjing Medical University Nanjing Jiangsu PR China

**Keywords:** CircRNA, colorectal cancer, translation, ubiquitination

## Abstract

**Background:**

The mitogen‐activated protein kinase (MAPK) pathway is highly associated with the progression and metastasis of various solid tumours. MAPK14, a core molecule of the MAPK pathway, plays vital roles in the colorectal cancer (CRC). Recent studies have shown that circRNAs can affect tumour progression by encoding peptides. However, little is known regarding the potential protein translated from circMAPK14 and whether it plays a role in the carcinogenesis of colorectal cancer.

**Methods:**

The RNA level and translatable potential of circMAPK14 in CRC was verified using qRT‐PCR and public databases. RNase R digestion assay, qRT‐PCR, sanger sequencing and FISH assays were utilised to verify the circular characteristics and subcellular localisation of circMAPK14. The suppressive role of circMAPK14 on the progression and metastasis of CRC was verified in vivo and in vitro. LC/MS analysis combined with western blotting demonstrated the presence and relative expression of circMAPK14‐175aa. The underlying mechanism of circMAPK14‐175aa action to inhibit CRC was identified by co‐IP analysis. The binding of U2AF2 within the flanking introns of circMAPK14 was evaluated by RNA pull‐down assay and RIP assay. Ultimately, luciferase reporter gene assays and ChIP assays confirmed that FOXC1 suppressed transcription of U2AF2 by binding to the U2AF2 promoter in the –400 bp to –100 bp region.

**Results:**

We identified that hsa_circ_0131663 (termed circMAPK14) showed significantly decreased expression level in cells and tissue samples of CRC, and was primarily localised in the cytoplasm. A series of function experiments demonstrated that circMAPK14 influenced CRC progression and metastasis by encoding a peptide of 175 amino acids (termed circMAPK14‐175aa). We also found that circMAPK14‐175aa reduced nuclear translocation of MAPK14 by competitively binding to MKK6, thus facilitating ubiquitin‐mediated degradation of FOXC1. Moreover, we described a positive feedback loop in CRC in which elevated FOXC1 expression was caused by reduced circMAPK14‐175aa expression. This, in turn, decreased circMAPK14 biogenesis by suppressing U2AF2 transcription.

**Conclusion:**

In summary, we reported for the first time that circMAPK14 functioned as a tumour‐suppressor by encoding circMAPK14‐175aa, which blocked the progression and metastasis of colorectal cancer.

## Graphical abstract

In summary, we first announced that circMAPK14 functions as a tumour‐suppressor through encoding circMAPK14‐175aa in the progression and metastasis of colorectal cancer.

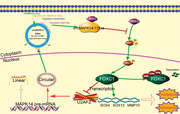

## 1 INTRODUCTION

Colorectal cancer (CRC) remains the third highest prevalence accompanying with the second highest cancer‐related mortality worldwide.^1^ Presently, CRC is commonly diagnosed at an advanced stage.^2^ Metastasis and recurrence remain the most critical causes of CRC‐related death, in spite of substantial improvements in treatment options.^3^, ^4^ Thus, it is urgent to explore the mechanism underlying the progression and metastasis of CRC in order to identify novel therapeutic targets.

The MAPK signalling pathway is one of many classical signal transduction pathways, which transport extracellular signals into a cell through receptors, G proteins, protein kinases, transcription factors and other signals, ultimately leading to cell proliferation, differentiation, apoptosis, metastasis etc.^5^ The clinical trials and molecular evidence indicated the importance of MAPK signalling pathways in CRC.^6^, ^7^ MAPK14 is the key members of the p38 MAPKs family, which plays a core function in the signalling cascade that ultimately activates transcription factors in response to an external stimulus.^8^ Gupta et al. reported a dual function of MAPK14 in colon cancer,^9^ and other studies have shown that MAPK14 was phosphorylated by the upstream map kinase kinase 3/6 (MKK3/6) to promote nuclear translocation.^10^ In summary, MAPK14 plays a vital role in colorectal cancer. However, there is no report on whether the circRNAs derived from MAPK14 is functional in CRC.

Previous studies have shown that circRNA, as a non‐coding RNA, could not be translated. However, recently published data suggest that the highly conserved open reading frames (ORFs) in circRNAs may encode specific functional peptides relied on the internal ribosome entry site (IRES),^11^, ^12^ m6A modification^13^ or rolling cycle amplification (RCA).^14^ In CRC, circRNAs also play important roles that involve translation of functional protein.^15^ More importantly, it remains to be determined whether circRNAs derived from MAPK14 genes are capable of translating novel peptides to promote CRC progression.

Here, in this study, we first identified the downregulation of circMAPK14 in CRC cell lines and tissues. Next, we found that circMAPK14 acted to suppress the progression and metastasis of CRC via encoding a novel protein (termed circMAPK14‐175aa). Further experiments confirmed that circMAPK14‐175aa promoted FOXC1 ubiquitination and degradation by competitively interacting with MKK6 as well as reducing nuclear translocation of MAPK14. Moreover, elevated FOXC1 expression in CRC was induced by a decrease in circMAPK14‐175aa, which in turn decreased the biogenesis of circMAPK14 through suppression of U2AF2 transcription. Taken together, these data revealed a positive feedback loop that reciprocally modulated the expression level and function of circMAPK14.

## 2 METHODS

### 2.1 Cell lines and tissues

All of human CRC specimens and adjacent non‐tumour tissues were surgically collected from the Department of General Surgery, First Affiliated Hospital of Nanjing Medical University. Patients received no chemotherapy or radiotherapy before surgery and signed informed consents. The research was approved by the Medical Ethics Committee of the First Affiliated Hospital of Nanjing Medical University. Human CRC cell lines (SW480, DLD‐1, LoVo, HT29, HCT116, Caco2) and normal human colon mucosal epithelial cell line (NCM460) were obtained from the Cell Center of Shanghai Institutes for Biological Sciences. Cells were cultured in Dulbecco's modified Eagle's medium (Gibico, USA) supplemented with 10% foetal bovine serum (Gibico, USA) and 1% penicillin/streptomycin (Gibico, USA) at 37°C with an atmosphere of 5% CO_2_ in a humidified cell chamber.

### 2.2 RNA extraction, qRT‐PCR, RNase R and actinomycin D assays

TRIzol reagent (Invitrogen, USA) was applied to isolate RNA from CRC cells and tissues samples. Genomic DNA (gDNA) was prepared from cells by PureLink Genomic DNA Mini Kit (Thermo Fisher Scientific, USA). A 7500 Real‐Time PCR System (Applied Biosystems, USA) was used to conducted qRT‐PCR analysis. We used GAPDH as endogenous control, and the relative primer sequence was listed in Table S1. Total RNA was incubated for 30 min with 3 U/mg of RNase R (Epicentre Technologies, USA), and the transcription was impeded by the addition of 2 mg/ml Actinomycin D. After the treatment, the relative RNA levels of MAPK14 and circMAPK14 was examined by qRT‐PCR. The experiment was performed in triplicate.

### 2.3 Sanger sequencing

We employed the sanger sequencing assay to detect the entire length of the amplification products. The back‐splice junction of hsa_circ_0131663 was identified by Sanger sequencing (Tsingke, Nanjing, China).

### 2.4 CCK‐8 assay

The cell proliferation ability was monitored by the Cell Counting Kit‐8 assay (Beyotime Biotechnology, Shanghai, China). A total of 1 × 10^3^ cells were cultured into 96‐well plates and measured absorbance values at 450 nm everyday by the automatic microplate reader (BioTek, Winooski, VT, USA). The experiment was performed in triplicate.

**HIGHLIGHTS**

- A novel protein (circMAPK14‐175aa), encoded by circMAPK14, exhibited tumour‐suppressive effects and competed with upstream kinase MKK6 to facilitate ubiquitin‐mediated degradation of FOXC1 and block CRC development.
- Reduction of FOXC1 expression increased U2AF2 transcript levels, which further facilitated the circularisation of circMAPK14.
- Taken together, our findings identified a novel circRNA that provided promising potential for improving therapeutic strategies in CRC.

### 2.5 Fluorescence in situ hybridisation (FISH) assay

The fluorescence‐labelled oligonucleotide probe complementary to circMAPK14 was designed by RiboBio, and the FISH analysis was conducted by utilised fluorescent In Situ Hybridisation Kit (RiboBio, Guangzhou, China) according to the manufacturer's protocols.

### 2.6 5‐Ethynyl‐2′‐deoxyuridine (EdU) assay

The treated cells were first plated into 96‐well plates (3 × 10^4^/well) and cultured for 24 h before the adjunction of EdU (50 μmol/L). Next, cells were fixed in 4% formaldehyde for 2 h and permeabilised with 0.5% TritonX‐100 for 10 min at room temperature. 1×Apollo reaction solution (400 μl) was added to react with the EdU (Beyotime Biotechnology, Shanghai, China) for 30 min, and DAPI (400 μl) was added for 30 min to stain the nucleus after washing with PBS for three times. Finally, a Nikon microscope (Nikon Japan) was used to capture the images of cells. The experiment was performed in triplicate.

### 2.7 Colony formation assay

The different transfection of cells were plated into 6‐well plates with complete medium to culture for 2 weeks. After that, the numbers of colony formation were counted after staining with crystal violet. The experiment was performed in triplicate.

### 2.8 Wound‐healing assay

We plated the stably transfected cells into 6‐well plates (1 × 10^6^ cells/well). After incubation for 24 h, we scratched the cell plates with a sterile 200 μl pipette tip along the middle of the well. The photographs were gained by microscope at 0 h and incubating for 48 h with the serum‐free medium. The experiment was performed in triplicate.

### 2.9 Colony formation assay

The different transfection of cells were plated into 6‐well plates with complete medium to culture for 2 weeks. After that, the numbers of colony formation were counted after staining with crystal violet. The experiment was performed in triplicate.

### 2.10 Transwell assay

A total of 4 × 10^4^/well transfected cells in 250 μl fresh medium with FBS were plated into the upper chamber including a Matrigel‐coated or uncoated membrane. After that, the complete medium (750 μl) was supplied to the lower section. The cells that invaded or migrated to the low membrane were stained with 0.1% crystal violet (Beyotime Biotechnology, Shanghai, China) after 48 h incubation. The experiment was performed in triplicate.

### 2.11 Western blotting assay

The BCA kit (Beyotime Biotechnology, Shanghai, China) was utilised to measure the concentration of protein was quantified by using. After the electrophoresis, the PVDF membranes were used. We incubated the membranes with different specific primary antibodies at 4°C overnight after blocking with 5% non‐fat milk at a shaker for 2 h. Another day, the corresponding secondary antibodies were used at room temperature for 2 h. Finally, the relative levels were calculated with a Bio‐Imaging System. The product numbers of antibodies were listed: MAPK14 (ab170099, Abcam, 1:1000), MKK6 (ab33866, Abcam, 1:1000), FOXC1 (ab226219, Abcam, 1:2000), p‐MAPK14 phosphorylate T180 + Y182 (ab195049, Abcam, 1:1000), SOX4 (ab70598, Abcam, 1:1000), SOX13 (ab96776, Abcam, 1:1000), MMP10 (ab261733, Abcam, 1:1000), U2AF2(ab37530, Abcam, 1:250), Tubulin (ab6046, Abcam, 1:500) was used as a internal control. H3 (#4499, CST, 1:2000) was used as a nucleus internal control. The experiment was performed in triplicate.

### 2.12 Mass spectrometry analysis

The related proteins were resolved by electrophoresis, and a specific band was excised. Digested proteins were subjected to the Orbitrap Velos Pro LC/MS system (Thermo Fisher Scientific, USA). The fragment spectra were used for checking the NCBI database with the Mascot search engine.

### 2.13 Dual‐luciferase reporter assay

The IRES wild‐type, IRES‐mut#1 and IRES‐mut#2 sequence was constructed into the luciferase reporter gene of the circMAPK14 plasmid in HCT116 cells. Then a dual‐luciferase reporter kit was carried out (Promega, WI, USA) according to the manufacturer's protocols. The experiment was performed in triplicate.

### 2.14 Co‐immunoprecipitation (Co‐IP)

The treatment cells after co‐transfection were lysed with IP lysis buffer (Beyotime Biotechnology, Shanghai, China). Flag beads (Sigma, USA) were added to the cell lysates at 4°C overnight. After that, the precipitates were detached by SDS‐PAGE. Then the protein was transferred onto the PVDF membrane and incubated with specific antibodies for determination.

### 2.15 RNA pull‐down assay

For the RNA binding protein (RBP)–intron interaction, the introns RNAs were obtained using in vitro transcription by T7 RNA polymerase (Ambion Life, USA) and purified by RNeasy Plus Mini Kit (QIAGEN, USA). Then, we conducted the pull down assay by the biotin‐labelled RNAs and Streptavidin magnetic beads (Life Technologies). Finally, western blotting assay was utilised to detect the proteins pulled‐down.

### 2.16 Animal models

In this study, all animal experiments were give official approval by The Nanjing Medical University Ethics Committee. The 5‐week‐old female BALB/c nude mice were used. To establish the tumour growth models, the stable transfected cells (1 × 10^6^ cells/100 μl) with PBS suspension were subcutaneously injected. To build the lung metastasis models, the distinct treated cells were injected into the tail vein of nude mice. After 6 weeks, we observed the occurrence of lung metastases and verified them by using H&E staining. To construct the liver metastasis models, we strictly follow the sterile principle to open the mouse abdomen after anaesthesia, and then injected 50 μl of 5 × 10^5^ stable transfected cells into the spleen. After the operation, we sutured the abdomen. Similarly, we assessed the liver metastasis ability after 6 weeks.

### 2.17 Statistical analysis

The data were presented as mean ± SD. We statistically analysed the data through Student's *t*‐test using GraphPad Prism 7. The association of circMAPK14 with clinicopathologic features was analysed by the χ^2^ test. Survival analysis was evaluated by Kaplan‐Meier plots. *p < .05, **p < .01 and ***p < .001. All experiments were performed in triplicate.

## 3 RESULTS

### 3.1 Characterisation of circMAPK14 in CRC cells and tissues

**FIGURE 1:**
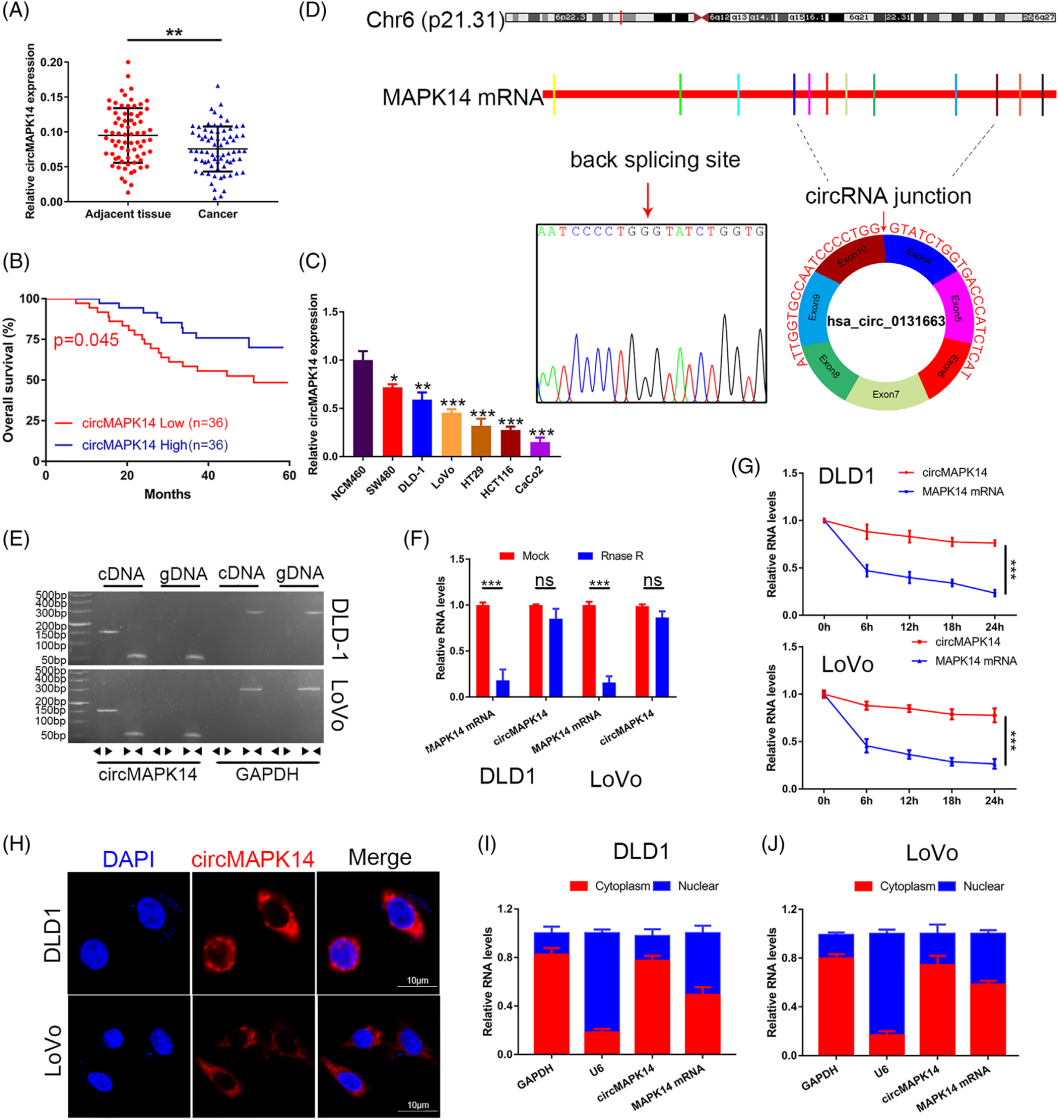
Identification and characterisation of circMAPK14 in CRC tissues and cell lines. (A) The relative expression of circMAPK14 in 72 CRC tissues and matched normal samples. (B) Kaplan–Meier plot analysis of correlations between the circMAPK14 levels and OS of 72 CRC patients. (C) The relative expression of circMAPK14 in CRC cell lines (SW480, DLD‐1, LoVo, HT29, HCT116, Caco2) compared with control (NCM460). (D) Sanger sequencing was utilised to confirm that circMAPK14 is generated from 4 to 10 exon of MAPK14 by head‐to‐tail splicing. (E) CircMAPK14 was only amplified via divergent primers in cDNA rather than gDNA by northern blot assay, GAPDH was used as a control. The relative levels of circMAPK14 and MAPK14 mRNA were detected after the treatment of (F) RNase R and (G) actinomycin D in DLD‐1 and LoVo cells. (H) The analysis of FISH indicated that circMAPK14 was mainly located in the cytoplasm. (I), (J) The qRT‐PCR analysis of cell fractions to certificate the subcellular localisation of circMAPK14 in DLD‐1 and LoVo cells

Fan et al. have demonstrated the critical role that the MAPK signalling pathway played in the progression of CRC.^5^ To determine the core importance of MAPK14 in CRC, we first analysed the levels of circRNAs derived from MAPK14 in CRC. The circRNAs hsa_circ_15030, hsa_circ_14100, hsa_circ_27976 and hsa_circ_24603 were identified in the circRNADb database. Of these, only hsa_circ_0131663 (hsa_circ_24603) was found to be significantly downregulated in 72 pairs of CRC samples compared with matched adjacent tissues by qRT‐PCR (Figure 1A); this circRNA was termed circMAPK14 in this study. The other three circRNAs showed no significant statistical differences in expression in CRC compared to normal tissues (Figure S1A‐C). We next explored the relationship between circMAPK14 RNA level and clinicopathological characteristics of CRC patients. The results indicated that a lower abundance of circMAPK14 was positively associated with tumour size, TNM stage and lymphatic metastasis (Table 1). Moreover, Kaplan–Meier curve survival analysis demonstrated worse overall survival in patients with lower expression of circMAPK14 (Figure 1B). We then determined the expression of circMAPK14 in six human CRC cell lines (SW480, DLD‐1, LoVo, HT29, HCT116 and Caco2) and found obviously decreased circMAPK14 expression in these cells compared to a normal human colon mucosal epithelial cell line (NCM460) (Figure 1C).

**TABLE 1:** Expression of circMAPK14 expression in human colorectal cancer according to patients'clinicopathological characteristics

		circMAPK14 expression		
Characteristics	Number	High group	Low group	*p* Value
Age (years)				
<60	21	10	11	.795
≥60	51	26	25	
Gender				
Male	37	17	20	.479
Female	35	19	16	
Size (cm)				
<5	36	23	13	.018*
≥5	36	13	23	
stage				
I/II	29	19	10	.03*
III/IV	43	17	26	
T stage				
T1/T2	33	18	15	.478
T3/T4	39	18	21	
N stage				
Absent (N0)	29	20	9	.008**
Present (N1–N3)	43	16	27	

* *p* < .05 statistically significant difference.

** *p* < 0.01.

The circMAPK14 is generated from exon 4 to exon 10 of the MAPK14 gene and has a full length of 536 nt. We performed Sanger sequencing to confirm the head‐to‐tail splicing of circMAPK14 (Figure 1D), and then designed the divergent and convergent primers to detect the circular form RNA and linear form mRNA, respectively. Agarose gel electrophoresis illustrated that the circMAPK14 was successfully detected by the convergent primers from gDNA and cDNA, but it was only generated from cDNA via divergent primers in DLD‐1 and LoVo cells (Figure 1E). To further verify the stability of circMAPK14, RNase R and actinomycin D were used. Compared with the linear form mRNA of MAPK14, circMAPK14 was resistant to RNase R digestion and had a longer half‐life (Figure 1F and G), which suggested that circMAPK14 was more stable than the linear form mRNA. Subsequently, FISH assays (Figure 1H) and qRT‐PCR analysis of cell fractions (Figure 1I and J) confirmed that circMAPK14 was predominantly localised to the cytoplasm and not the nucleus.

### 3.2 The circMAPK14 suppresses proliferation and migration of CRC cells

**FIGURE 2:**
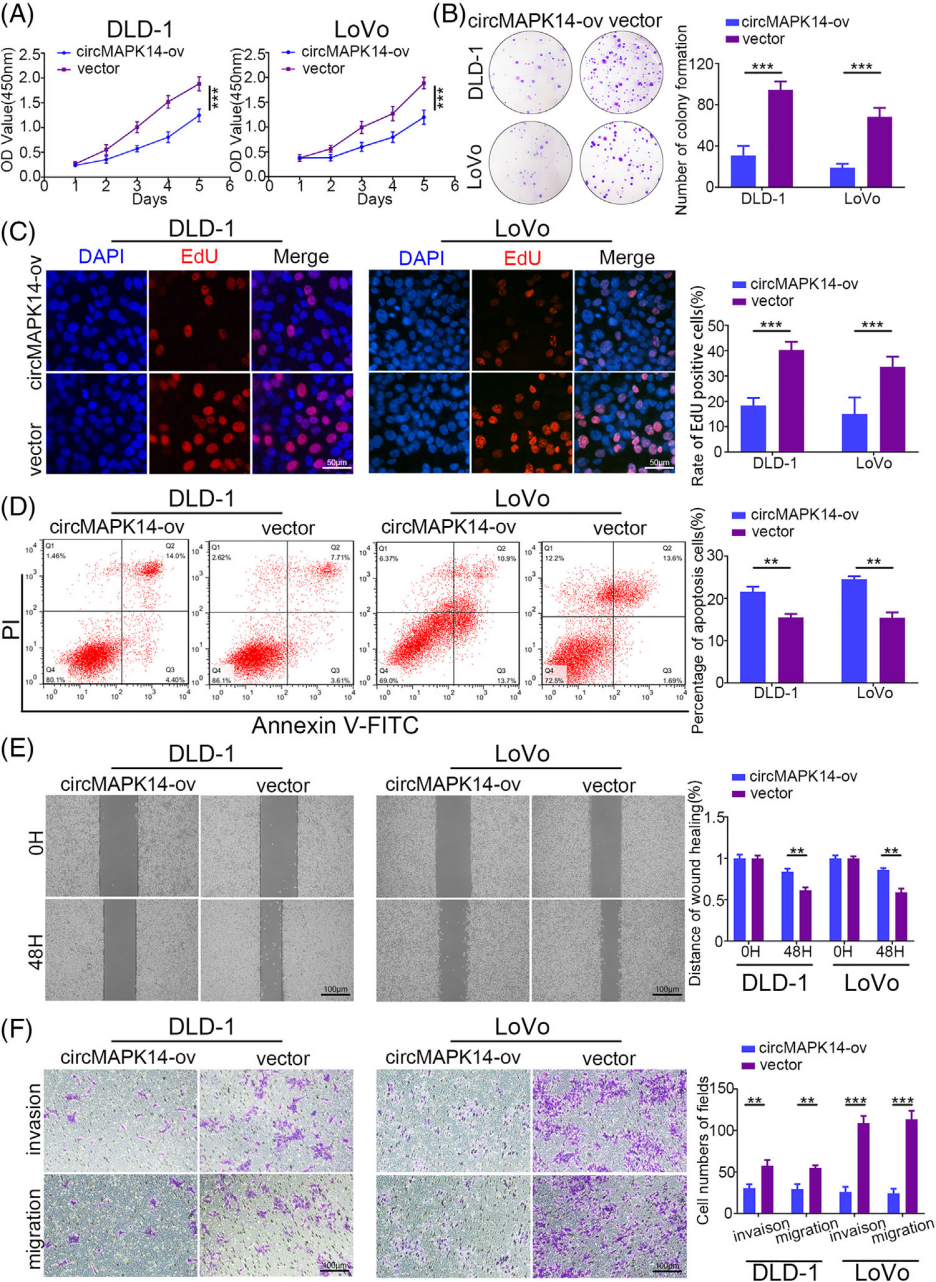
CircMAPK14 inhibits the proliferation and migration of CRC cells. The effect of circMAPK14 on CRC proliferation was evaluated by (A) CCK8 assay, (B) colony formation assay and (C) EdU incorporation assay. (D) The flow cytometry assay was used to assess the apoptosis rate of CRC cells after altering the expression of circMAPK14. The effect of circMAPK14 on CRC invasion and migration was examined by (E) wound healing assay and (F) Transwell assay

To probe the biological functions of circMAPK14 in CRC cells, we constructed lentiviral overexpression vectors (circMAPK14‐ov) and three lentiviral knockdown vectors (circMAPK14‐sh), avoiding the off‐target influence (Figure S1D). After evaluating transfection efficiencies by qRT‐PCR, we selected circMAPK14‐ov and circMAPK14‐sh#1 for subsequent function experiments because these constructs specifically regulated the expression of circMAPK14 rather than that of MAPK14 mRNA (Figure S1E and F). As expected, analysis through CCK8 assay (Figure 2A), colony formation assay (Figure 2B) and EdU incorporation assay (Figure 2C) indicated that the proliferation ability of CRC cells was markedly weakened upon upregulation of circMAPK14. On the contrary, cell viability (Figure S2A), clonality (Figure S2B) and EdU incorporation rate (Figure S2C) were markedly enhanced when circMAPK14 was silenced. A subsequent flow cytometry analysis was carried out to evaluate the rate of cell apoptosis. We found that increased circMAPK14 expression resulted in an elevated apoptosis rate in CRC cells (Figure 2D), whereas knockdown of circMAPK14 group had opposite effect (Figure S2D). Upon completing wound healing and Transwell assays, we observed that the invasion and migration abilities of CRC cells were dramatically inhibited following transfection with circMAPK14‐ov (Figure 2E and F). Conversely, the counter events were observed in the suppression of circMAPK14 (Figure S2E and F).

### 3.3 The circMAPK14 encodes a novel protein in CRC

**FIGURE 3:**
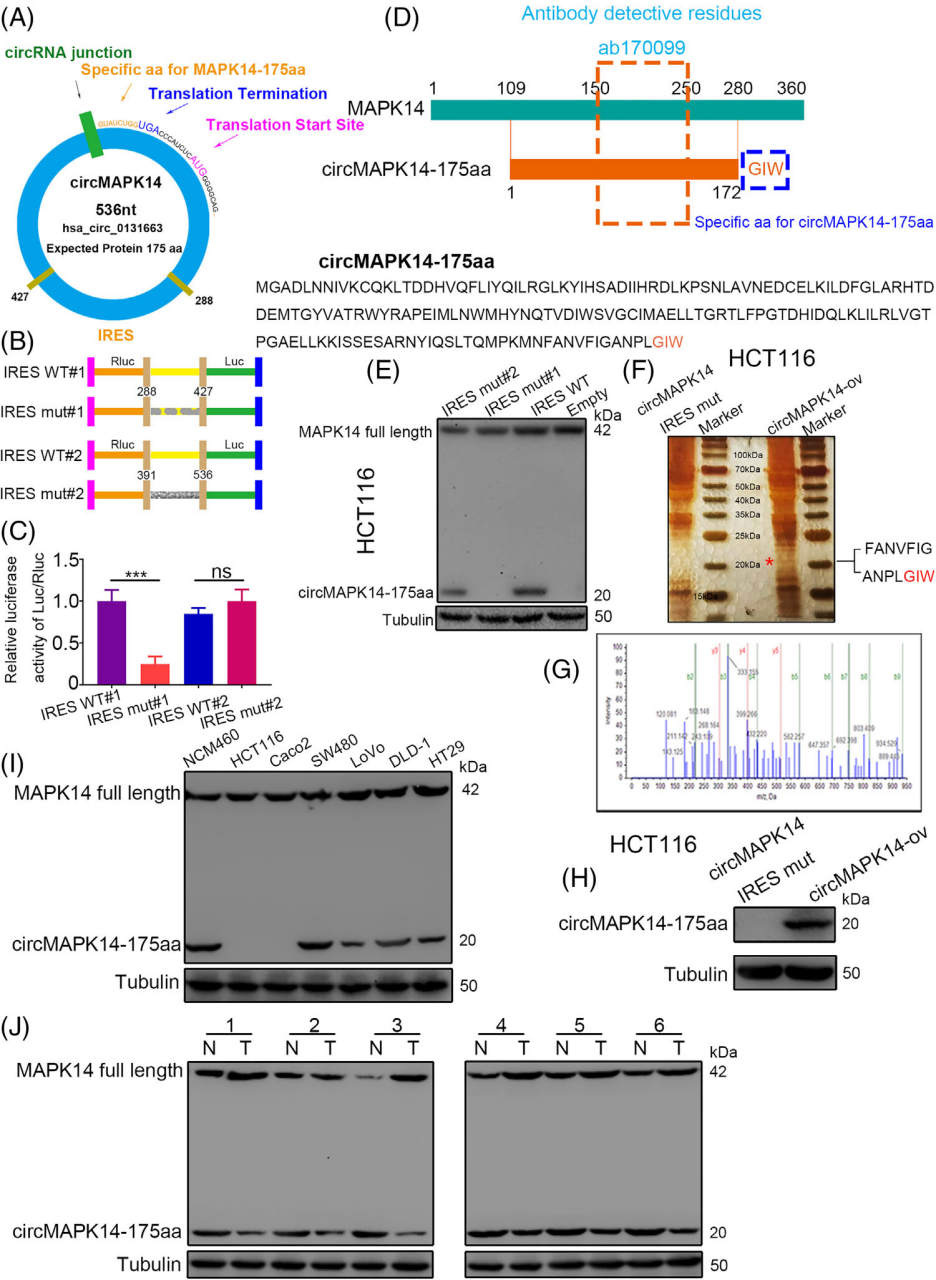
CircMAPK14 encodes a novel protein of 175 amino acids, termed as circMAPK14‐175aa. (A) The translation potential of circMAPK14 was showed in the schematic diagram. (B) The wild‐type or two mutant of IRES sequence were cloned between Rluc and Luc reporter genes. (C) The activity of IRES sequence was tested by dual luciferase reporter assay. (D) The description of circMAPK14‐174aa sequence and MAPK14 sequence and the antibody (ab170099) recognised the same residues of them. (E) The activity of IRES sequence was verified by western blotting assay. (F) The total protein of HCT116 cell transfected with circMAPK14‐ov and IRES‐mut#1 was separated by electrophoresis and sliver staining. (G) Cut the corresponding gel band of prediction to perform LC‐MS/MS analysis. (H) The existence of circMAPK14‐175aa was revealed by western blotting assay. The expression of MAPK14 and circMAPK14‐175aa was determined in CRC (I) tissues and (J) cell lines

Upon analysis of the predicted results from the circRNADb database, we discovered that circMAPK14 contained an open reading frame (ORF) and a putative IRES sequence, potentially encoding a 175 amino acid protein (termed circMAPK14‐175aa in this study) (Figure 3A). We then verified the activity of these two IRES sequences (nt 288–427 and 391–536) in CRC (Figure 3B). We discovered that the luciferase activity of the IRES mut#1 reporter rather than that of the IRES mut#2 was obviously dropped compared with that of the wild‐type IRES reporter (Figure 3C). The 175 aa peptide covers a homologous sequence with aa 109–280 of MAPK14, but has a special C‐terminal ‘Gly Ile Trp’ sequence. We found an antibody against the middle segment of MAPK14 (aa 150–250), which recognised both MAPK14 and circMAPK14‐175aa (Figure 3D). To further examine the activity of the IRES sequences, a western blot assay was conducted, which showed that circMAPK14 encoded a 175 aa protein with a molecular weight of 20 kDa driven by the IRES sequences at nucleotides (nt) 288–427 (Figure 3E). Next, we transfected IRES mut#1 and circMAPK14‐ov into HCT116 cells. The results of sliver staining (Figure 3F) and LC‐MS/MS analysis (Figure 3G) showed a sequence of FANVFIGANPLGIW, which was consistent with the previously identified 175 amino acid sequence containing a specific C‐terminal GIW sequence. We observed the same result by western blot analysis (Figure 3H). These findings confirmed that a 175 aa peptide was translated from circMAPK14. In CRC cell lines (Figure 3I) and tissues (Figure 3J), we found that the 175 aa peptide was expressed in significantly lower abundance compared to normal cell lines and tissues. We also found that circMAPK14‐ov and circMAPK14‐sh regulated the abundance of the 175 aa peptide, but did not affect MAPK14 protein expression in NCM460 cells (Figure S3A), DLD‐1 cells (Figure S3B and D) or LoVo cells (Figure S3C and E).

### 3.4 The circMAPK14 suppresses the CRC malignant phenotype in vitro via circMAPK14‐175aa

**FIGURE 4:**
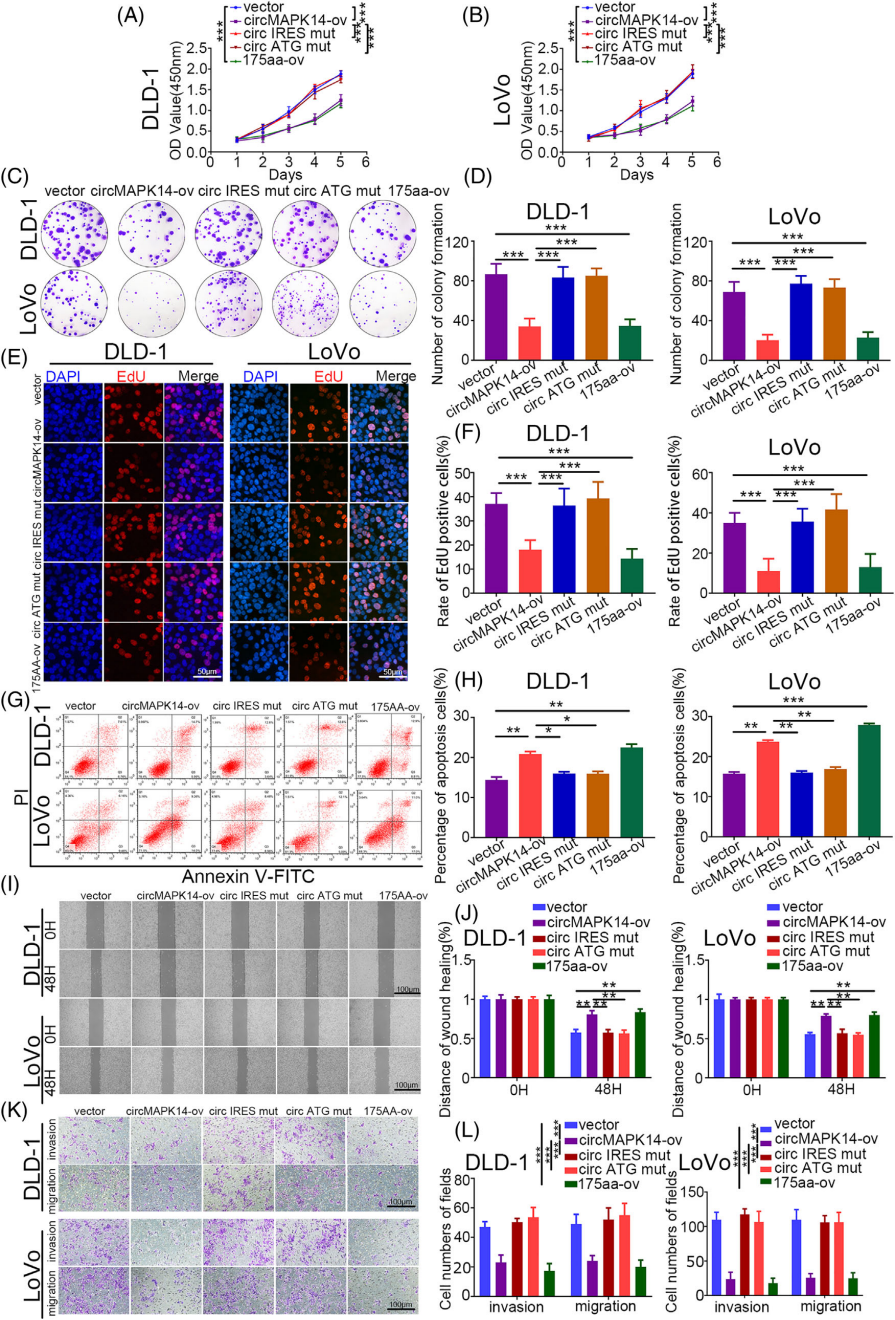
CircMAPK14 suppresses the CRC malignant phenotype via 175aa in vitro. After transfection of circMAPK14‐ov, circMAPK14 ATG mut, circMAPK14 IRES mut#1 and linearised 175aa‐ov in DLD‐1 and LoVo cells, the abilities of proliferation were evaluated by (A) and (B) CCK8 assay, (C) and (D) colony formation assay, (E) and (F) EdU incorporation assay. (G), (H) The apoptosis rates were evaluated by flow cytometry assay. The abilities of invasion and migration were evaluated by (I) and (J) wound healing assay, (K) and (L) Transwell assay

To further evaluate the biological effects of circMAPK14 in CRC, we transfected circMAPK14‐ov, circMAPK14 ATG mut, circMAPK14 IRES mut, linearised 175aa‐ov and circMAPK14‐sh#1 into DLD‐1 and LoVo cell lines. Following transfection with circMAPK14‐ov and linearised 175aa‐ov, circMAPK‐175aa expression was increased compared to empty vector control; there was no significant difference after transfection with circMAPK14 ATG mut or circMAPK14 IRES mut (Figure S3C and D). Inversely, circMAPK‐175aa expression was reduced following transfection with circMAPK14‐sh#1 (Figure S3E and F). As mentioned previously, the expression of the linear form of MAPK14 did not change. CCK8 assays (Figure 4A and B), colony formation assays (Figure 4C and D), and EdU incorporation assays (Figure 4E and F) illustrated that circMAPK14 obviously repressed the proliferative capacity of CRC cells via increased expression of circMAPK‐175aa; linearised 175aa‐ov rescued the promotional effects of the proliferation capacity by transfecting circMAPK14‐sh (Figure S4A–F). We found that the apoptosis rate of CRC cells was increased upon transfection with circMAPK14‐ov and 175aa‐ov, but not circMAPK14 ATG mut or circMAPK14 IRES mut (Figure 4G and H). In contrast, the knockdown group exhibited decreased apoptosis, which was reversed by linearised 175aa‐ov (Figure S4G and H). Subsequent wound healing assays demonstrated that circMAPK14 inhibited the migration ability of CRC cell lines via circMAPK‐175aa (Figures 4I, J and S4I, J), which is also consistent with results of Transwell assays (Figures 4K, L and S4K, L).

### 3.5 The circMAPK14 attenuates tumourigenicity and metastasis of CRC in vivo via circMAPK14‐175aa

**FIGURE 5:**
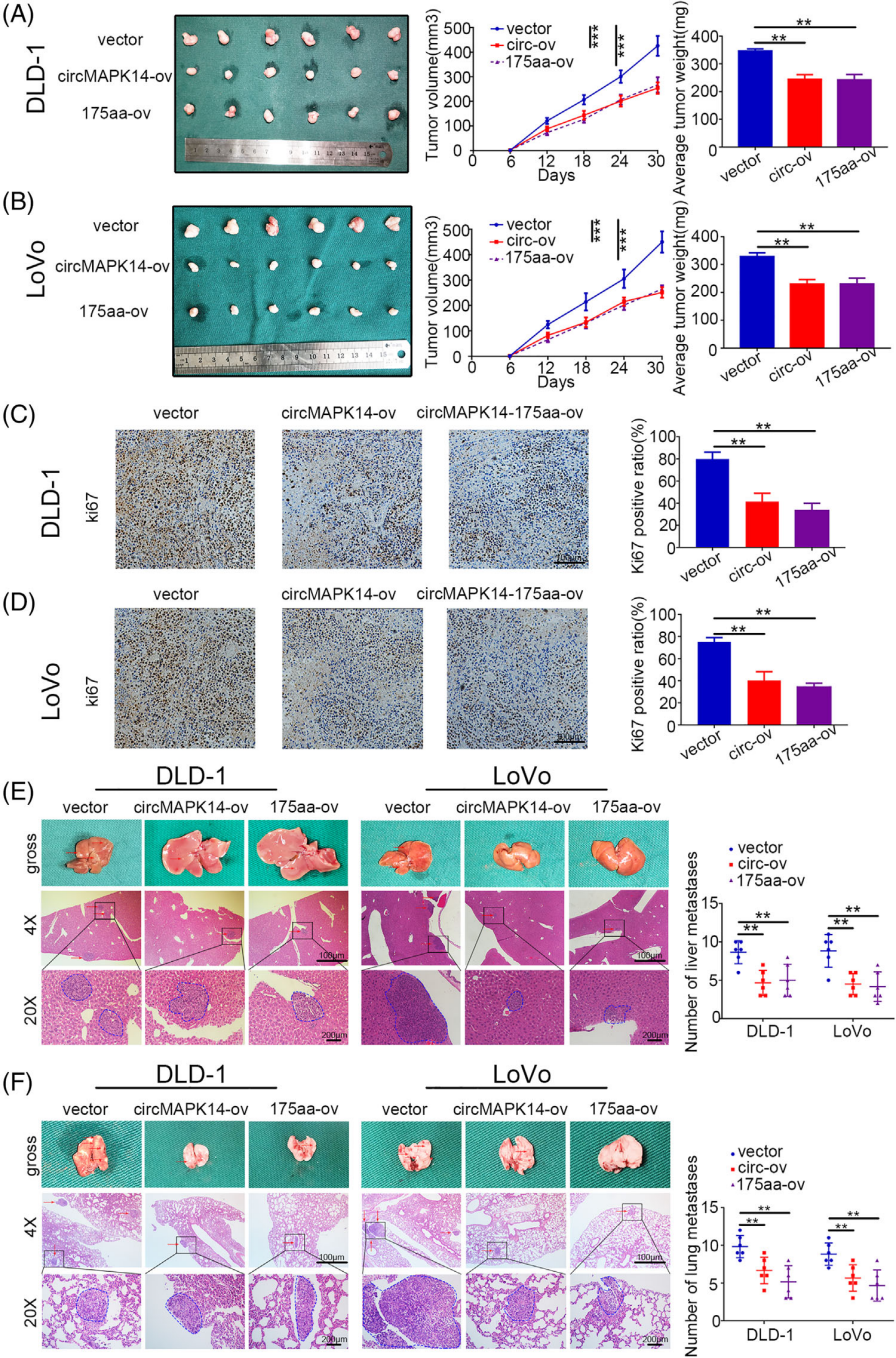
CircMAPK14 attenuated tumourigenicity and metastasis of CRC via 175aa in vivo. (A), (B) The above cells were injected subcutaneously into nude mice to assess the suppressive function of circMAPK14‐175aa. In vivo tumourigenicity was monitored, and mice were sacrificed after 30 days. (C), (D) The xenograft tumours were stained with ki67. (E) The above cells were injected into the tail vein of nude mice to assess the suppressive effect on lung metastasis of circMAPK14‐175aa. Mice lung were subjected to H&E staining. (F) The above cells were injected into the spleen of nude mice to assess the suppressive effect on liver metastasis of circMAPK14‐175aa. Mice liver were subjected to H&E staining

To further verify our in vitro findings, we developed a subcutaneous tumour model, a lung metastasis model and a liver metastasis model in mice. First, HCT116 cells were transfected with the various constructs and injected subcutaneously into nude mice. Then we observed the status of tumourigenesis every 6 days. Consistent with our previous results, circMAPK14 restrained tumourigenesis through translation of a 175aa peptide that was dependent upon an IRES sequence at nt 288–427 (Figure S5A). Moreover, circMAPK14 weakened tumour growth by increasing circMAPK14‐175aa expression (Figure 5A and B). Silencing of circMAPK14 accelerated tumour growth, and this was blocked by 175aa‐ov (Figure S5B and C). We then obtained the xenograft tumours to conduct Ki67 staining as a measure of proliferation. Tumour samples with high levels of circMAPK14 exhibited weaker Ki67 staining (Figure 5C and D), whereas circMAPK14 knockdown tumour samples showed stronger Ki67 staining (Figure S5D and E). Furthermore, we utilised metastasis models to determine the function of circMAPK14 on regulating tumour metastasis. We found a significant alleviation of the lung metastasis locus with upregulation of circMAPK14 and circMAPK14‐175aa (Figure 5E), while adverse tumour events were observed with stable knockdown of circMAPK14. While circMAPK14‐175aa rescued this influence (Figure S5F). A similar phenomenon was observed with live metastasis models (Figures 5F and S5G). Collectively, these data further supported that circMAPK14 encoded a 175aa peptide that functioned to attenuate tumourigenesis and metastasis in CRC.

### 3.6 The circMAPK14‐175aa represses MAPK14 phosphorylation by competitively binding to MKK6

**FIGURE 6:**
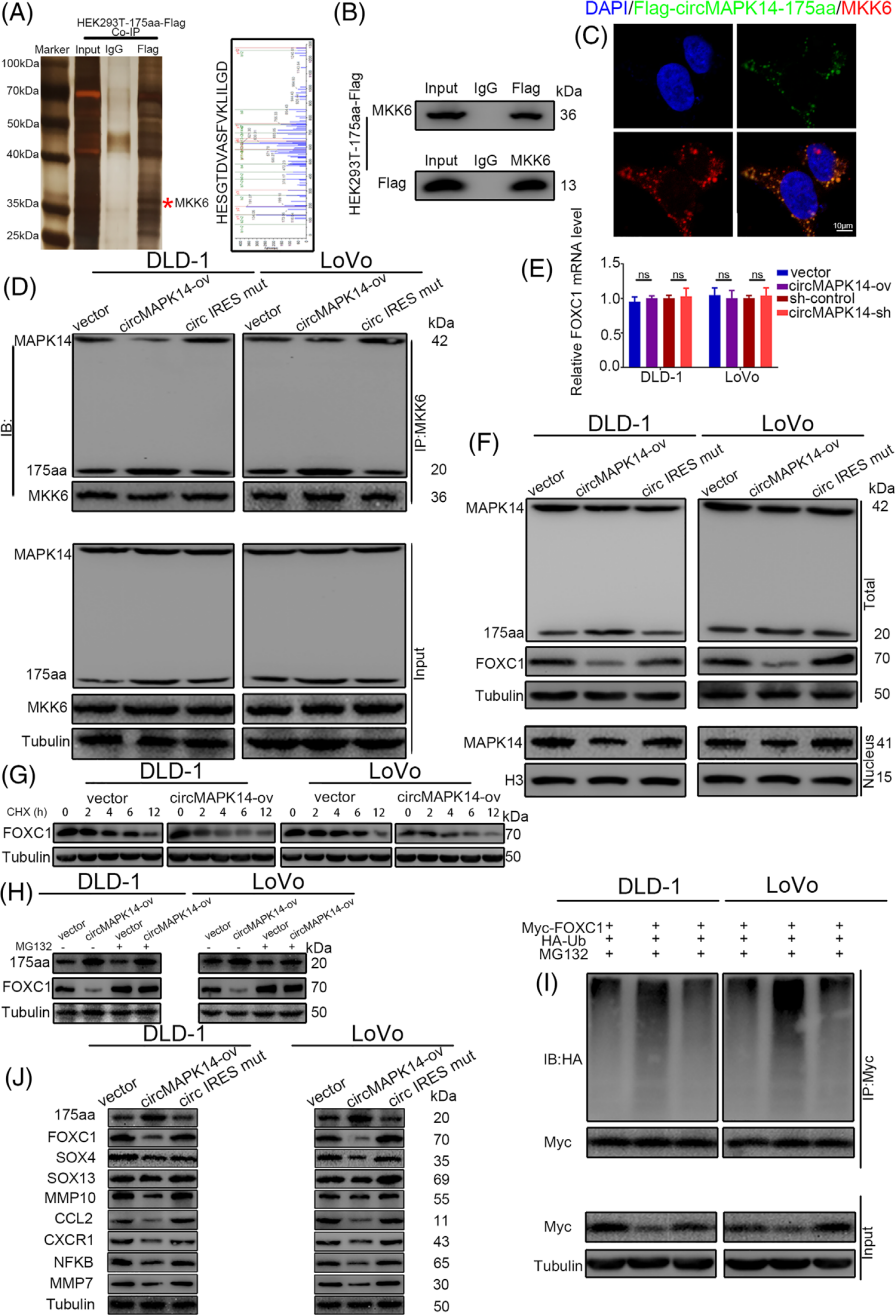
CircMAPK14‐175aa represses the phosphorylation of MAPK14 by competing with MKK6 and subsequently promotes the degradation of FOXC1 via ubiquitination. (A) LEFT panel, total protein from Flag‐circMAPK14‐175aa transfected HEK293T cells were separated via SDS‐PAGE. Right panel, MKK6 was identified by LC‐MS analysis. (B) Mutual interaction of Flag‐circMAPK14‐175aa and MKK6 were confirmed by Co‐IP assay. (C) Immunofluorescence colocalisation between Flag‐circMAPK14‐175aa and MKK6. Blue represent DAPI, green represent Flag‐circMAPK14‐175aa, red represent MKK6. (D) When the level of 175aa was rose, the amount of MAPK14 but not 175aa binding with MKK6 was apparently reduced by Co‐IP assay against MKK6 antibody. (E) The relative expression of FOXC1 mRNA was detected by qRT‐PCR after alteration of 175aa level. (F) Upper panel, the relative level of FOXC1 protein was detected by western blotting assay after alteration of 175aa level. Lower panel, the relative level of p‐MAPK14 in the nucleus was detected by WB assay after alteration of 175aa level. (G) The time‐course experiment with WB assay was conducted after the treatment with cycloheximide (CHX, an inhibitor of protein synthesis). The results of WB assay showed that the ubiquitin‐proteasome system (UPS) played major roles in the degradation of FOXC1 after the treatment with (H) MG132 (a proteasome inhibitor). (I) Overexpression of circMAPK14 facilitated the FOXC1 degradation via ubiquitination. (J) The downstream target genes (SOX4, SOX13 and MMP10) levels were correspondingly changed along with the degradation of FOXC1

To explore the underlying mechanism influenced by circMAPK14‐175aa in CRC cells, we conducted immunoprecipitation (IP) experiments in HEK293T cells transfected with Flag‐tagged circMAPK14‐175aa. We identified MKK6 as a potential interacting protein in IP lysate by mass spectrometry (Figure 6A). Flag‐labelled circMAPK14‐175aa mutually interacted with MKK6 in HEK293T cells (Figure 6B). Immunofluorescence experiments displayed co‐localisation of circMAPK14‐175aa and MKK6 (Figure 6C).

A previous study reported that the activated upstream kinase MKK6 phosphorylated MAPK14 at phosphorylation sites Thr‐180 and Tyr‐182.^16^ When MAPK14 was activated via phosphorylation, it would transport from the cytoplasm into the nucleus to block degradation of FOXC1 by ubiquitination.^17^ Because circMAPK14‐175aa shares identical sequences with MAPK14 in aa 109–280 and also contains the same phosphorylation sites as MAPK14, we hypothesised that circMAPK14‐175aa would competitively binding to MKK6. The reciprocal IP assay indicated that circMAPK14 cannot directly bind with MKK6 (Figure S11B). Therefore, we performed co‐IP experiments using anti‐MKK6 antibody and illustrated that upregulation of circMAPK14 increased the degree to which circMAPK14‐175aa bound to MKK6, but reduced the degree to which MAPK14 bound to MKK6 (Figure 6D). In contrast, the knockdown group displayed the opposite results (Figure S6A). Moreover, there was no significant change in IRES mut group compared to empty vector control, suggesting that circMAPK14‐175aa abundance was not altered by IRES sequence mutation. The phosphorylation level of MAPK14 was verified by western blot assay in Figure S10B. All the data demonstrated that The circMAPK14‐175aa represses MAPK14 phosphorylation by competitively binding to MKK6.

### 3.7 The circMAPK14‐175aa promotes the degradation of FOXC1 via ubiquitination

Because our data thus far showed that MAPK14 interacted with forkhead box C1 (FOXC1) and blocked the degradation of FOXC1 by ubiquitination, we next detected the mRNA (Figure 6E) and protein levels (Figures 6F and S6B) of FOXC1 after alteration of circMAPK14 expression. As expected, the protein level of FOXC1 changed along with circMAPK14 alteration, but FOXC1 mRNA was unchanged. Furthermore, nuclear expression of MAPK14 was negatively correlated with circMAPK14 alteration by western blot assay (Figures 6F and S6B) and IF staining (Figure S11A). Moreover, circMAPK14 overexpression repressed the interaction between MAPK14 and FOXC1, while circMAPK14 inhibition increased this interaction. However, no obvious interaction was observed between circMAPK14‐175aa and FOXC1 (Figure S8), suggesting that circMAPK14‐175aa regulated FOXC1 expression by suppressing nuclear translocation of MAPK14 and not through direct binding to FOXC1.

To investigate whether circMAPK14 affected the stability of FOXC1, we treated CRC cells with cycloheximide (CHX, an inhibitor of protein synthesis) and completed a time‐course experiment (Figures 6G and S6C–E). We found that circMAPK14 promoted the degradation of FOXC1 protein, shortening the half‐life of FOXC1. The ubiquitin‐proteasome system (UPS) and the lysosomal system are the main proteolytic processes responsible for intracellular protein degradation.^18^ To determine the degradation pattern of FOXC1, we treated DLD‐1 and LoVo cells with MG132 (a proteasome inhibitor) (Figures 6H and S6F) or chloroquine (CHQ, a lysosome inhibitor) (Figure S11D) following stable transfection. These findings suggested that FOXC1 was degraded mainly via the UPS in CRC cells. To determine whether decreased circMAPK14 expression functioned to sustain the protein stability of FOXC1 by repressing ubiquitination, Myc‐FOXC1 and HA‐Ub were co‐transfected into DLD‐1 and LoVo cells with stable transfection of circMAPK14, and co‐IP assays were completed anti‐Myc. In accordance with our previous findings, overexpression of circMAPK14 enhanced ubiquitination of FOXC1 (Figure 6I), whereas silencing of circMAPK14 reduced the level of ubiquitination (Figure S6G). Furthermore, downstream target genes of FOXC1 also changed accordingly, including SRY‐box transcription factor 4 (SOX4), SOX13 and matrix metallopeptidase 10 (MMP10) (Figures 6J and S6H). Overall, these data suggested that circMAPK14‐175aa, which was encoded by circMAPK14, promoted FOXC1 degradation through inhibition of MAPK14 nuclear translocation and not via direct binding to FOXC1.

### 3.8 The circMAPK14‐175aa inhibits malignant biological behavior of CRC cells in vitro via FOXC1

To further validate whether circMAPK14 affected the malignant phenotype of CRC cells via regulation of FOXC1, we designed a series of rescue experiments. We stably co‐transfected circMAPK14‐ov with FOXC1‐ov and circMAPK14‐sh with FOXC1‐sh. CCK8 and colony formation assay results demonstrated that FOXC1 reversed the inhibitory function of circMAPK14 on CRC cell proliferation (Figure S7A and B) and colony formation (Figure S7C and D). Reduced FOXC1 expression eliminated the ability of circMAPK14‐sh to promote invasion and migration in CRC cells, as evaluated by wound healing assays (Figure S7E) and Transwell assays (Figure S7F). Moreover, Western blot analysis demonstrated that circMAPK14 deactivated downstream target genes (SOX4, SOX13, MMP10, CCL2, CXCR1, NFkB, MMP7) by suppressing the expression of FOXC1 (Figure S7I and J).

### 3.9 U2AF2 is a target of FOXC1 that facilitates circularisation of circMAPK14

Previous studies have reported that the back‐splicing process of circRNAs was catalysed by the spliceosome via interaction with complementary sequences in the flanking introns.^19^, ^20^ Several circRNAs are synthesised by competing with the production of linear isoforms.^21^ Thus, we postulated that a certain splicing factor must promote the biogenesis of circMAPK14. U2AF2 was predicted to bind to intron 3 and intron 10 of MAPK14 pre‐mRNA by MEME Suite (Figure S9A). To further verify the role of U2AF2 in regulating circMAPK14 biogenesis, we performed an RNA pull‐down assay and a RNA immunoprecipitation (RIP) assay. We found that U2AF2 bound to intron 3 and intron 10 of MAPK14 pre‐mRNA (Figure S9B–D). Furthermore, as shown in Figure S9E, U2AF2 overexpression led to increased circMAPK14 expression. We also constructed several vectors (Figure S9F) and detected the expression of circMAPK14 by qRT‐PCR after co‐transfection with U2AF2‐ov. Marked overexpression of circMAPK14 was observed in vectors containing intron 3 and intron 10 (Figure S9G), implying that the flanking introns were essential for the looping of circMAPK14 induced by U2AF2 (Figure S9G). Intriguingly, we noticed that U2AF2 expression was negatively correlated with that of FOXC1, according to TCGA database (Figure S9H). We observed a decrease in U2AF2 expression with increased FOXC1 expression (Figure S9I). Berry et al. reported that FOXC1 contained multiple functional domains that functioned in transcriptional regulation.^22^ Hence, we suspected that FOXC1 might transcriptionally repress the expression of U2AF2. As predicted by the JASPAR database, three putative binding regions (BR 1–3) were divided in U2AF2 promoter sequence and three primers (Primer 1–3) were designed correspondingly for these regions (Figure S9J). In combination with luciferase reporter gene assays (Figure S9K), ChIP assays (Figure S9L and M) and EMSA assay (Figure S10A), we illustrated that FOXC1 suppressed the transcription of U2AF2 by binding to the U2AF2 promoter in the ‐400 bp to ‐100 bp (BR 3) region.

**FIGURE 7:**
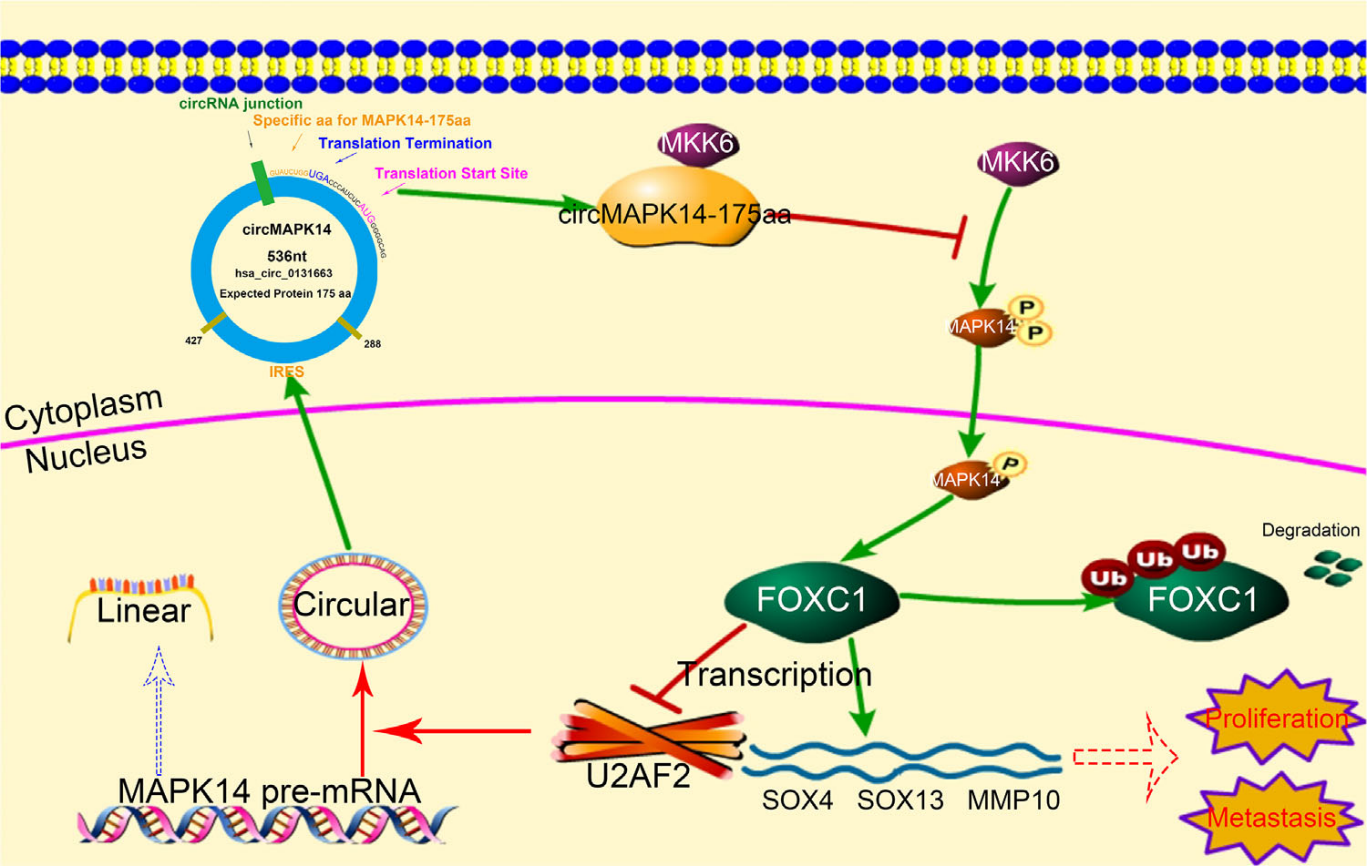
The mechanism diagram of circMAPK14‐175aa encoded by circMAPK14 on inhibiting tumourigenicity and metastasis of CRC by competing with MKK6

In summary, our data demonstrated that circMAPK14‐175aa was encoded by circMAPK14 and was dependent upon its ORF and IRES sequence. The circMAPK14‐175aa inhibited the phosphorylation of MAPK14 by competitively binding to MKK6, which led to the suppression of MAPK14 nuclear translocation. Furthermore, circMAPK14‐175aa accelerated the ubiquitin‐mediated degradation of FOXC1 and acted to suppress CRC progression and metastasis. Moreover, elevated FOXC1 in CRC was caused by reduced circMAPK14‐175aa expression. In turn, this decreased the circularisation efficiency of circMAPK14 by suppressing U2AF2 transcription, forming a positive feedback loop to regulate circMAPK14 in CRC (Figure 7). Our study suggested that this positive feedback loop may provide novel prospect for the advancement of therapeutic strategies for colorectal cancer.

## 4 DISCUSSION

The circRNAs are a novel class of non‐coding RNAs and have a covalently closed loop structure. They play crucial roles in many human diseases,^23^ including gastrointestinal malignancies.^24^, ^25^ Given their stability and conservation, circRNAs hold tremendous potential value as biomarkers and therapeutic targets.^24^ Most previous reports focused on the ability of circRNAs to impact disease by acting as miRNA sponges or through interaction with RBP.^26^, ^27^ Interestingly, several studies have reported that some artificial circRNAs can be translated in eukaryotic cells.^28^, ^29^ Translation of the circβ‐catenin encourages hepatocellular carcinoma (HCC) growth through activation of the Wnt pathway,^30^ and the comprehensive translation of circRNAs can be driven by N(6)‐methyladenosine.^13^ Gao et al. uncovered the critical role of secretory E‐cadherin protein variant, encoded by circE‐cadherin on stimulating of EGFR signalling.^31^ In our study, we first verified the circular characteristics and subcellular distribution of circMAPK14. Then, by utilising qRT‐PCR in combination with the circRNADb database, we realised the translation potential of circMAPK14 in CRC cells. Hereafter, we initially determined the impact of a new suppressive protein encoded by circMAPK14 on the progression and metastasis of CRC.

The MAPK pathway is well‐reported to integrate different signals in cancer development that affect proliferation, differentiation and migration,^32^, ^33^ and is particularly important in colorectal cancer.^5^ MAPK14 can be activated via dual phosphorylation of Thr‐180 and Tyr‐182 by MKK3 or MKK6.^16^ When MAPK14 is activated, it is translocated into the nucleus where it can act on downstream targets. Zhang et al. reported that MAPK14‐mediated FOXC1 stability was required for CRC metastasis.^17^ We observed that circMAPK14‐175aa possessed a similar sequence as its host gene, MAPK14. Thus, we assumed that this novel peptide, although shorter than its isoforms, would possess analogous roles. Accordingly, we used co‐IP assays and western blot analysis to demonstrate that circMAPK14‐175aa hindered nuclear translocation of MAPK14 by competing with MKK6. Then, because of circMAPK1414‐175aa, the sequel of blocking FOXC1 ubiquitination degradation relied on MAPK14 was moderated, which consecutively altered the level of downstream targets such as SOX4, SOX13 and MMP10.

More importantly, recent studies have reported that spliceosomes were involved in circRNA biogenesis, indicating competition between canonical splicing and back‐splicing.^21^, ^34^ In the present study, we predicted and confirmed that U2AF2 interacted with the flanking introns of MAPK14 pre‐mRNA to facilitate the circularisation of circMAPK14. We observed that MAPK14 linear form mRNA expression was negatively correlated with that of U2AF2, according to TCGA data (data not shown). Furthermore, U2AF2 was verified to be a transcriptional target of FOXC1, forming a positive feedback loop.

In summary, this study focused on the coding capacity of circular RNA via the IRES sequence. Some specific circRNAs have been reported to be translated by rolling‐cycle amplification^14^ and, in the future, we plan to explore more possibilities for circRNA translation. Because circRNAs have the potential to serve as novel diagnostic biomarkers of cancer,^35^ it would be beneficial to detect the levels of circMAPK14 and circMAPK14‐175aa in blood samples in the future. In the present study, we concentrated on FOXC1, which is one of the molecules regulated by MAPK14; this scope was relatively limited because the MAPK pathway is an extremely complex signalling network. Recent reports have shown that FOXC1 influenced CRC development through modulation of cisplatin resistance and glucose metabolism in CRC cells. Our future studies will aim to explore the role of circMAPK14‐175aa in these contexts.^36^, ^37^ Taken together, our findings highlighted a clear anti‐tumourigenic role for circMAPK14‐175aa, and additional studies are required to determine its usefulness in clinical application.

## 5 CONCLUSION

Taken together circMAPK14 is a newly identified MAPK14 transcriptional variant in our study. We discovered that circMAPK14 was downregulated in CRC, and that it was primarily located in the cytoplasm of CRC cells. Furthermore, a novel protein (circMAPK14‐175aa), encoded by circMAPK14, exhibited tumour‐suppressive effects and competed with upstream kinase MKK6 to facilitate ubiquitin‐mediated degradation of FOXC1 and block CRC development. Reduction of FOXC1 expression increased U2AF2 transcript levels, which further facilitated the circularisation of circMAPK14. Taken together, our findings identified a novel circRNA that provided promising potential for improving therapeutic strategies in CRC.

## CONFLICT OF INTEREST

The authors declare no conflict of interest.

## Supporting information

SUPPORTING INFORMATION

SUPPORTING INFORMATION

SUPPORTING INFORMATION

SUPPORTING INFORMATION

SUPPORTING INFORMATION

SUPPORTING INFORMATION

SUPPORTING INFORMATION

SUPPORTING INFORMATION

SUPPORTING INFORMATION

SUPPORTING INFORMATION

SUPPORTING INFORMATION

SUPPORTING INFORMATION

SUPPORTING INFORMATION

SUPPORTING INFORMATION

SUPPORTING INFORMATION

SUPPORTING INFORMATION
