# Supplementary figures and images for: A novel tumour suppressor protein encoded by circMAPK14 inhibits progression and metastasis of colorectal cancer by competitively binding to MKK6
Source: Clin Transl Med. 2021 Oct 14;11(10):e613. doi: 10.1002/ctm2.613 (PMC8516360; doi:10.1002/ctm2.613)

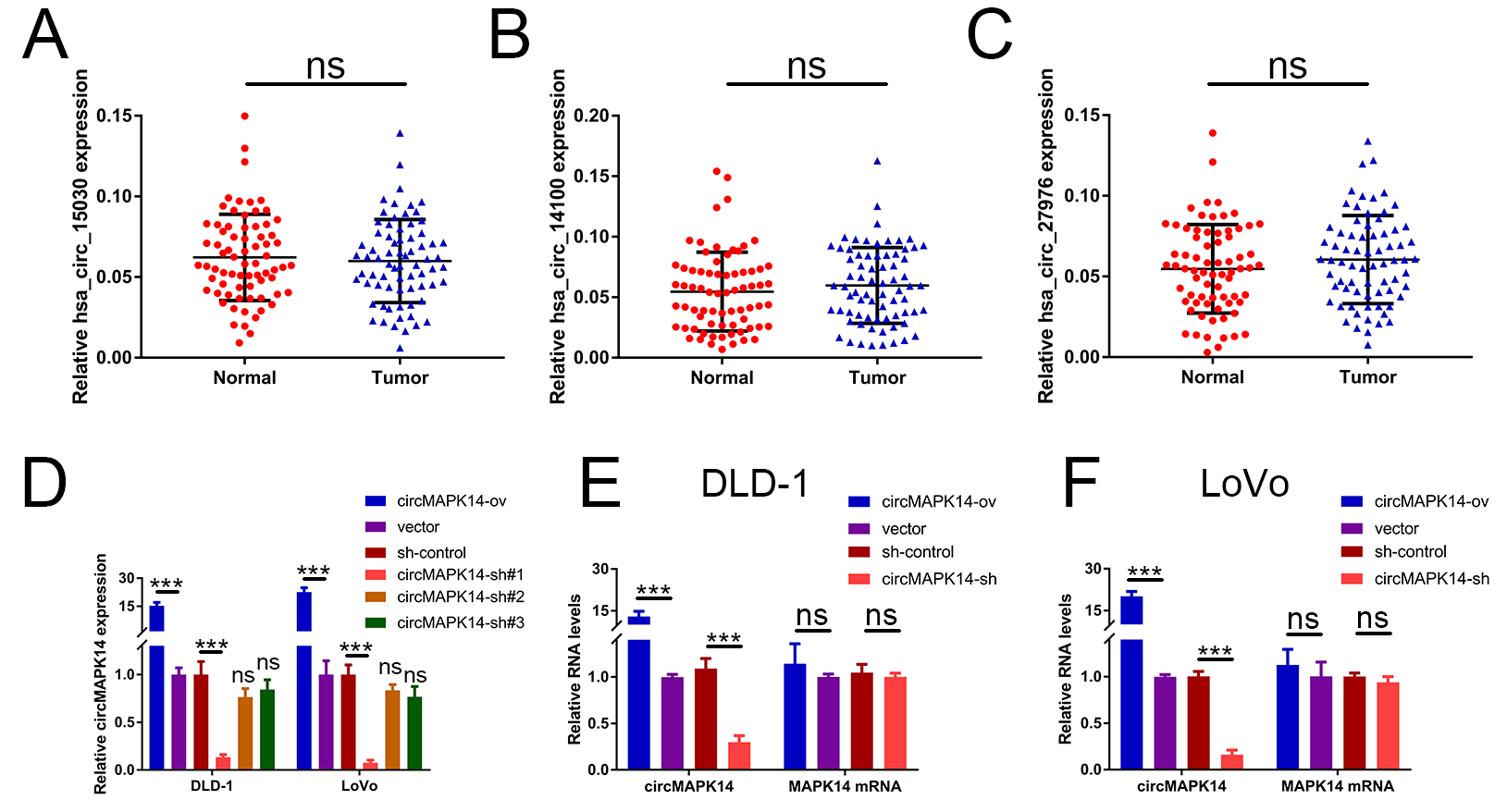

Supplement: Supplementary file 1 — SUPPORTING INFORMATION [file CTM2-11-e613-s008.tif]

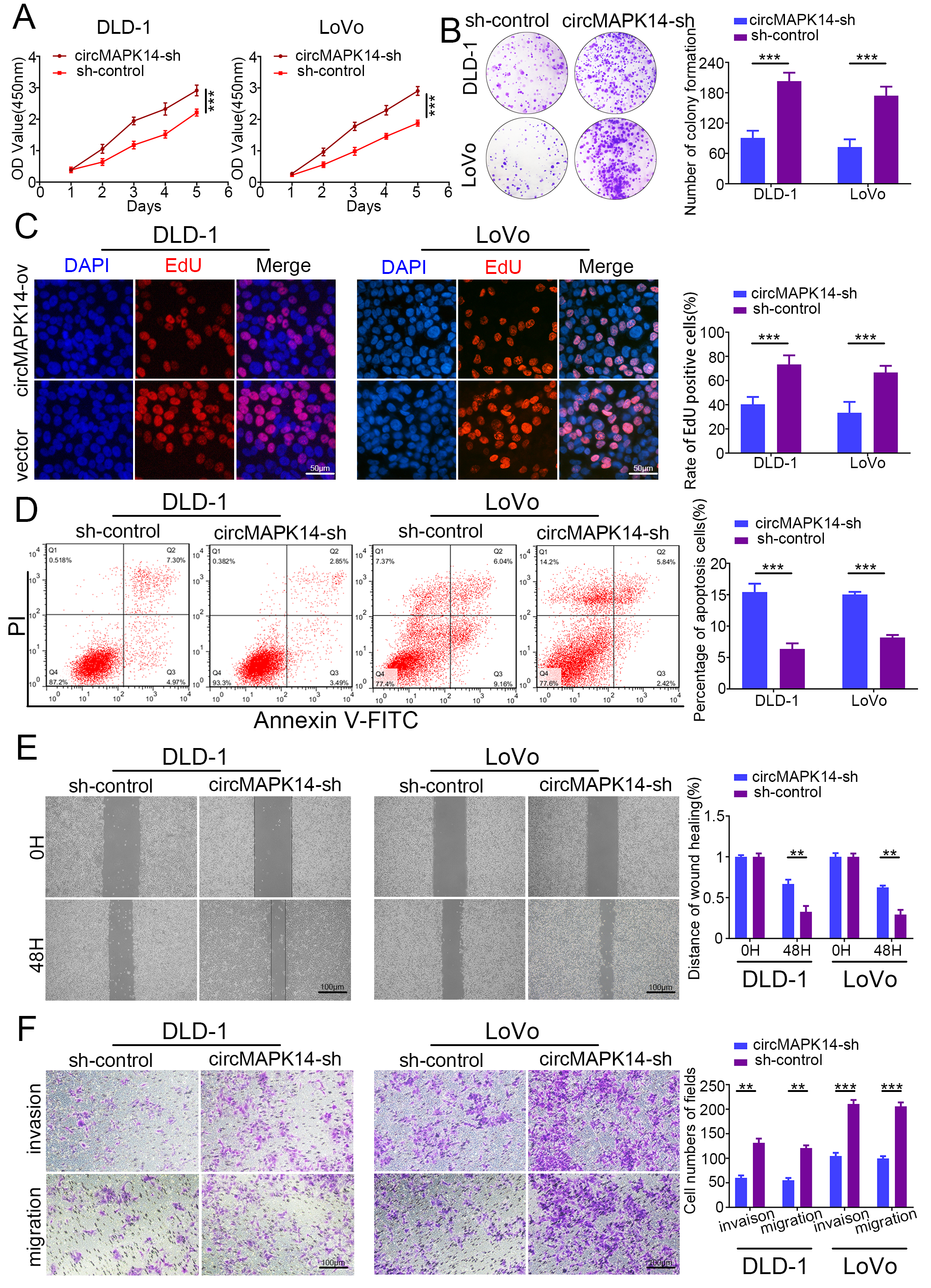

Supplement: Supplementary file 2 — SUPPORTING INFORMATION [file CTM2-11-e613-s002.tif]

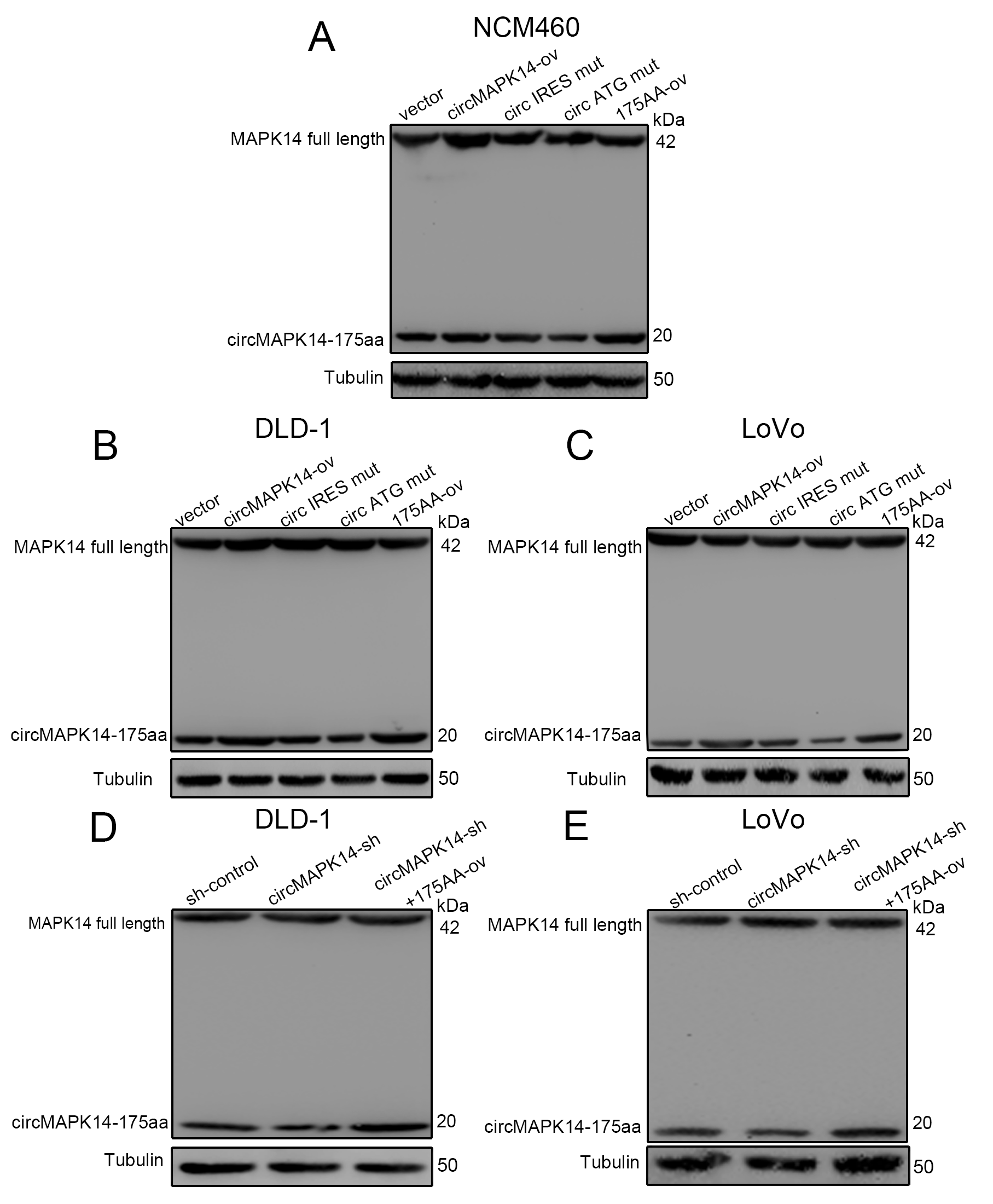

Supplement: Supplementary file 3 — SUPPORTING INFORMATION [file CTM2-11-e613-s016.tif]

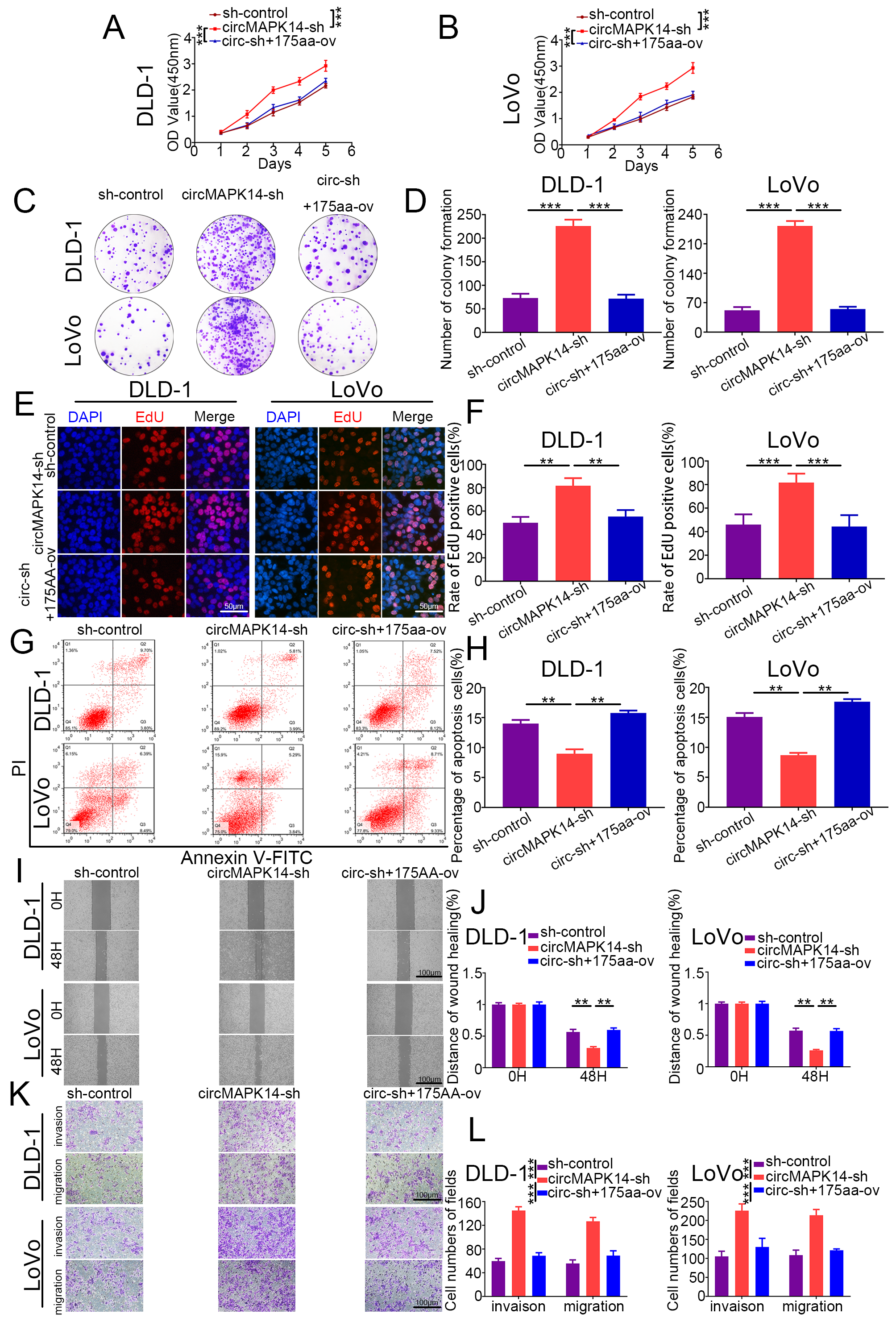

Supplement: Supplementary file 4 — SUPPORTING INFORMATION [file CTM2-11-e613-s011.tif]

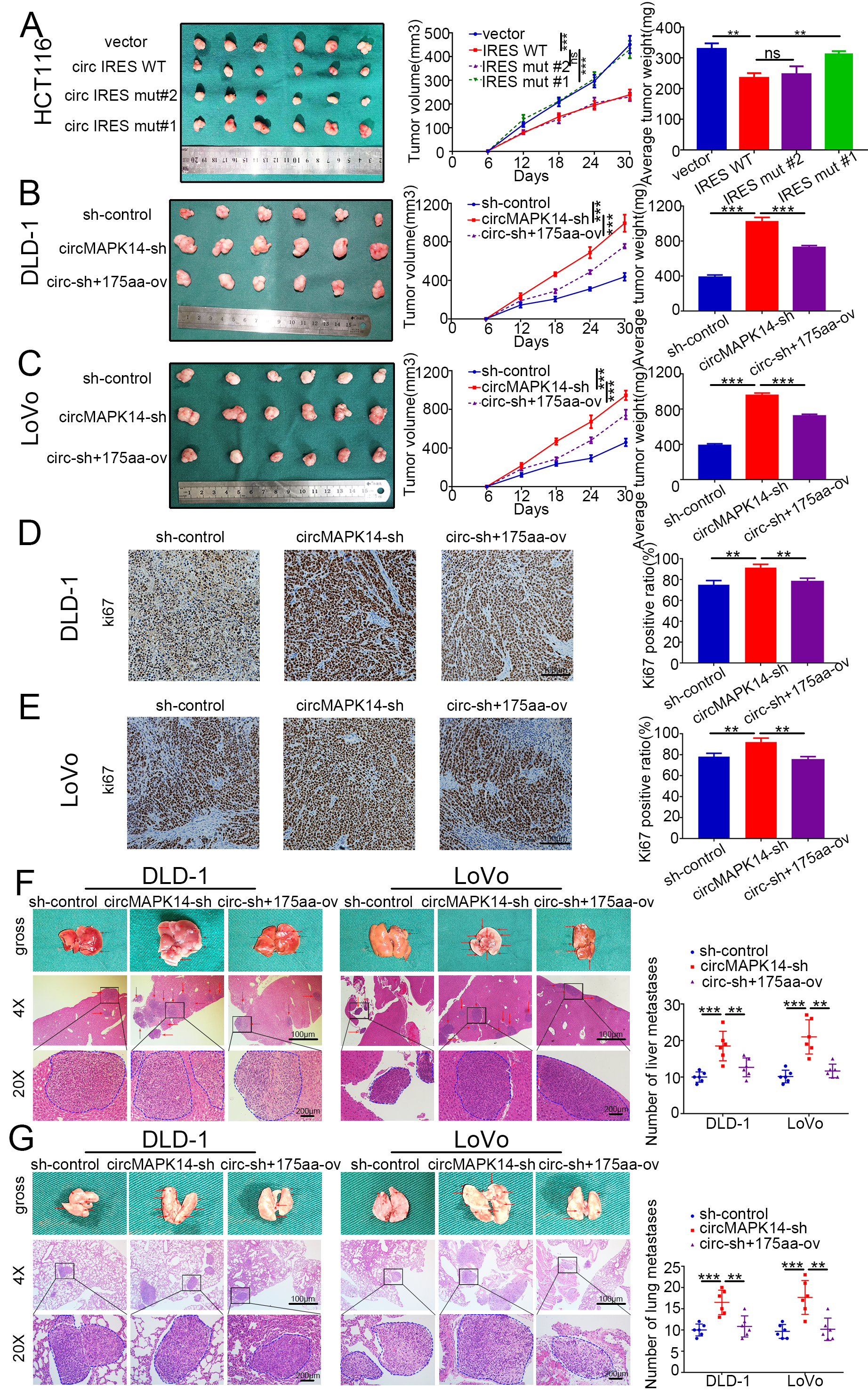

Supplement: Supplementary file 5 — SUPPORTING INFORMATION [file CTM2-11-e613-s006.tif]

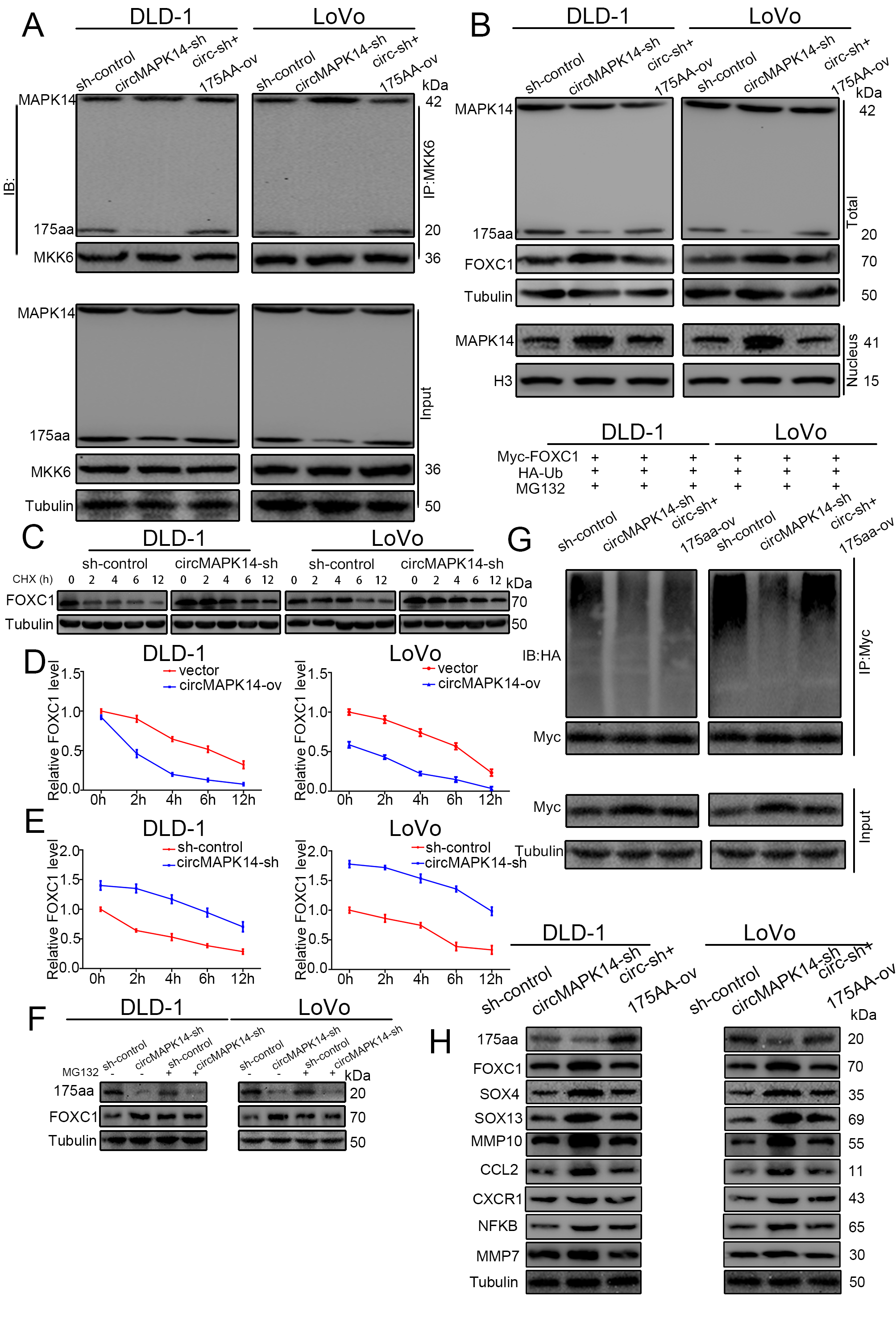

Supplement: Supplementary file 6 — SUPPORTING INFORMATION [file CTM2-11-e613-s013.tif]

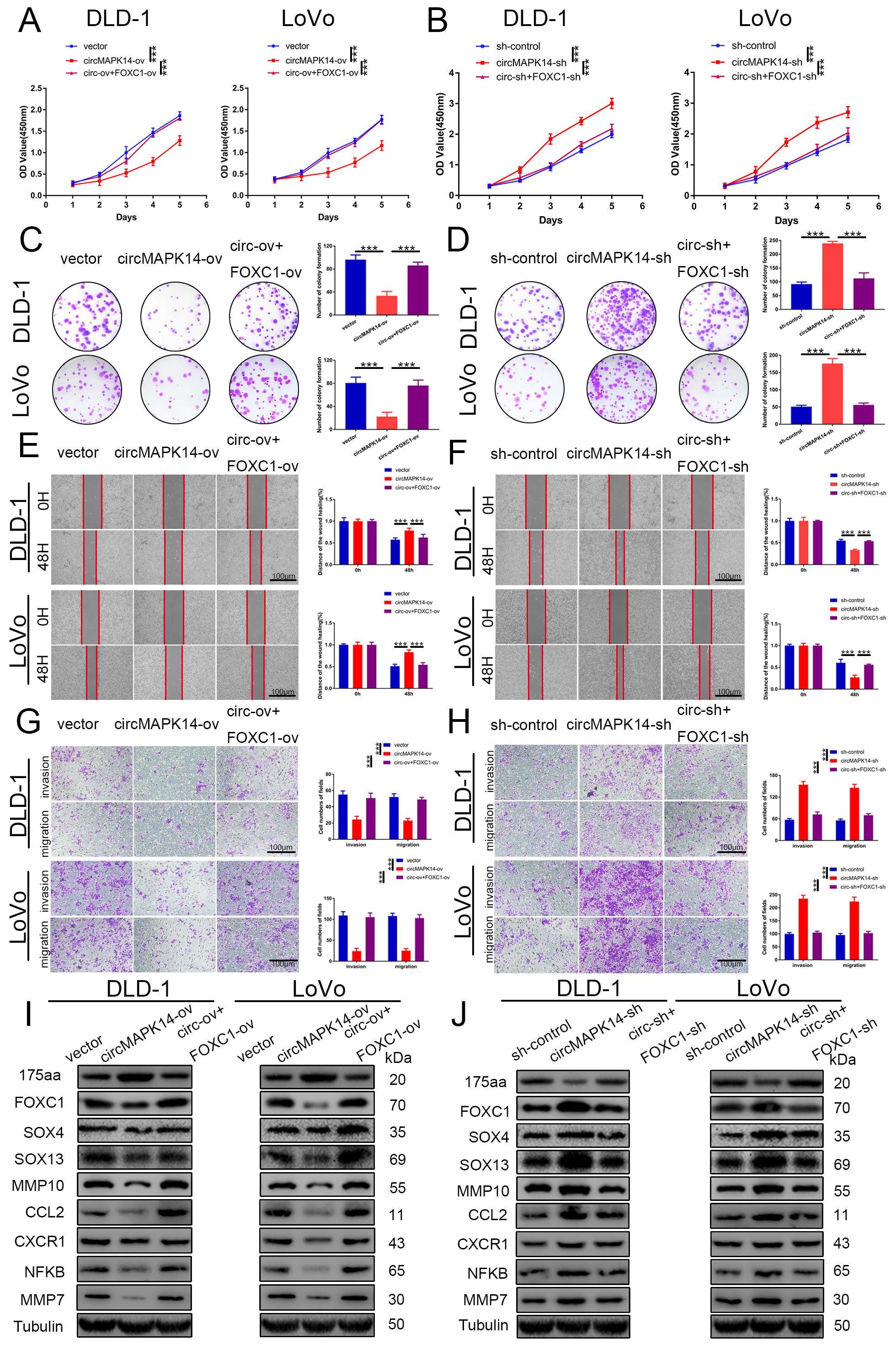

Supplement: Supplementary file 7 — SUPPORTING INFORMATION [file CTM2-11-e613-s015.tif]

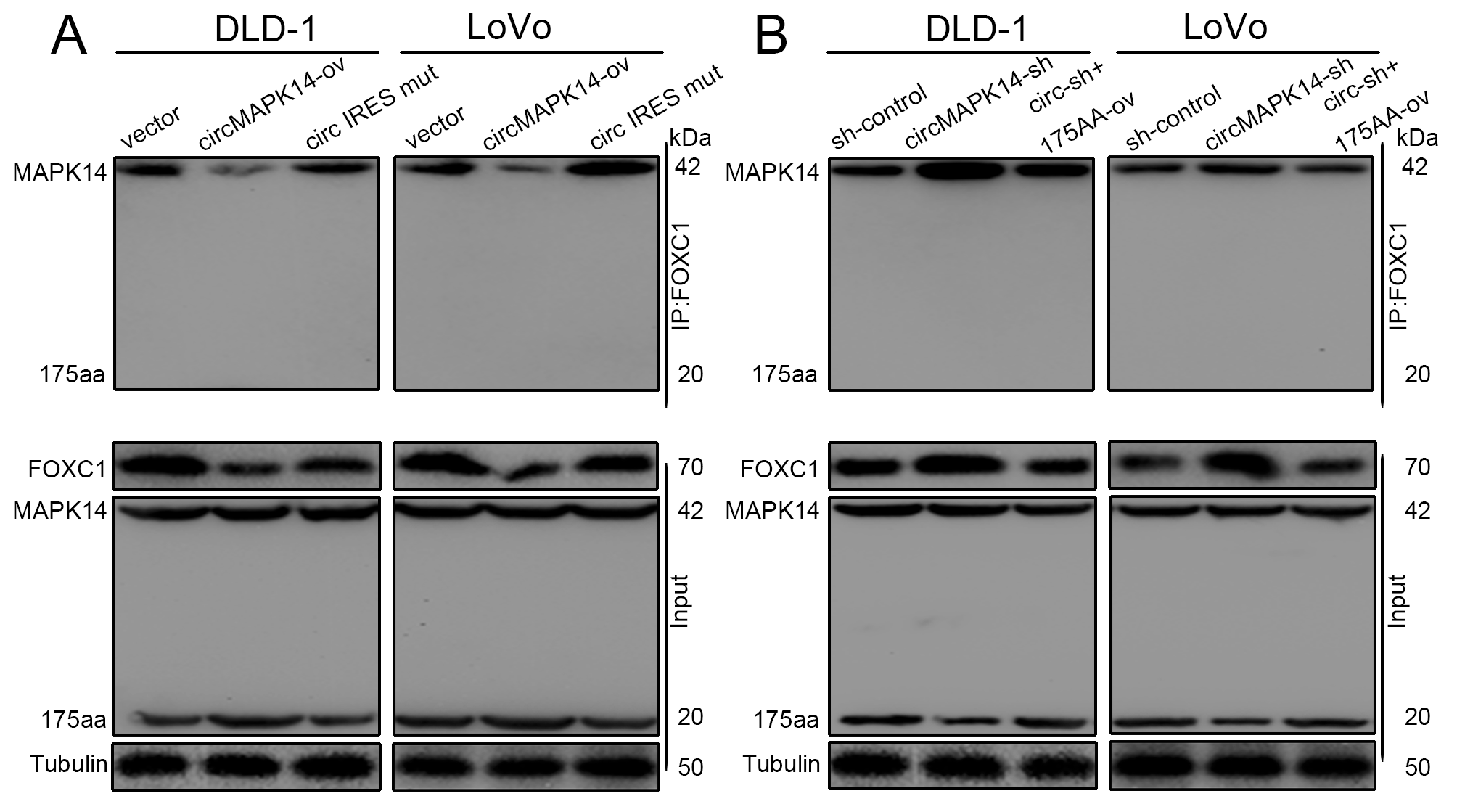

Supplement: Supplementary file 8 — SUPPORTING INFORMATION [file CTM2-11-e613-s009.tif]

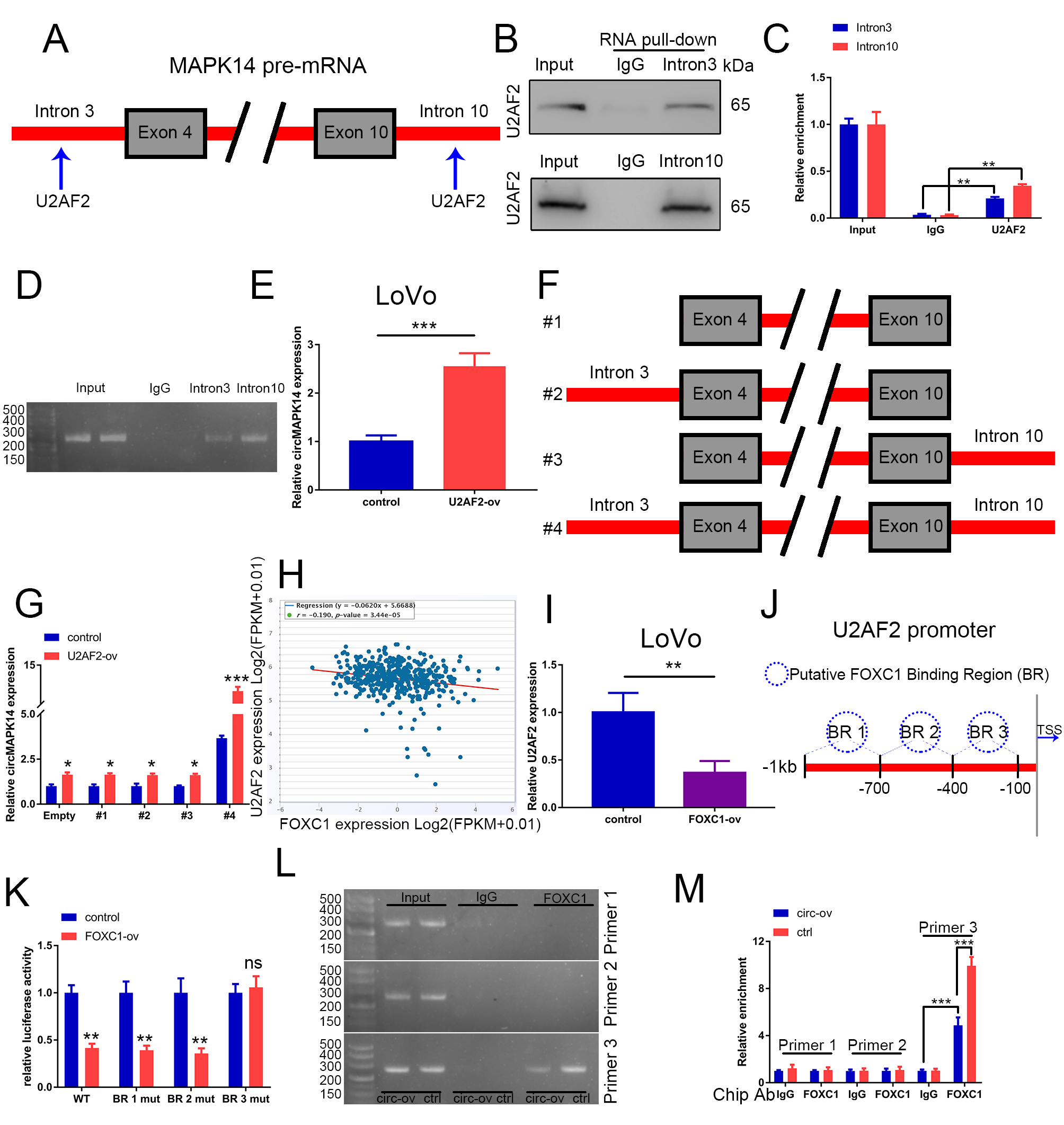

Supplement: Supplementary file 9 — SUPPORTING INFORMATION [file CTM2-11-e613-s003.tif]

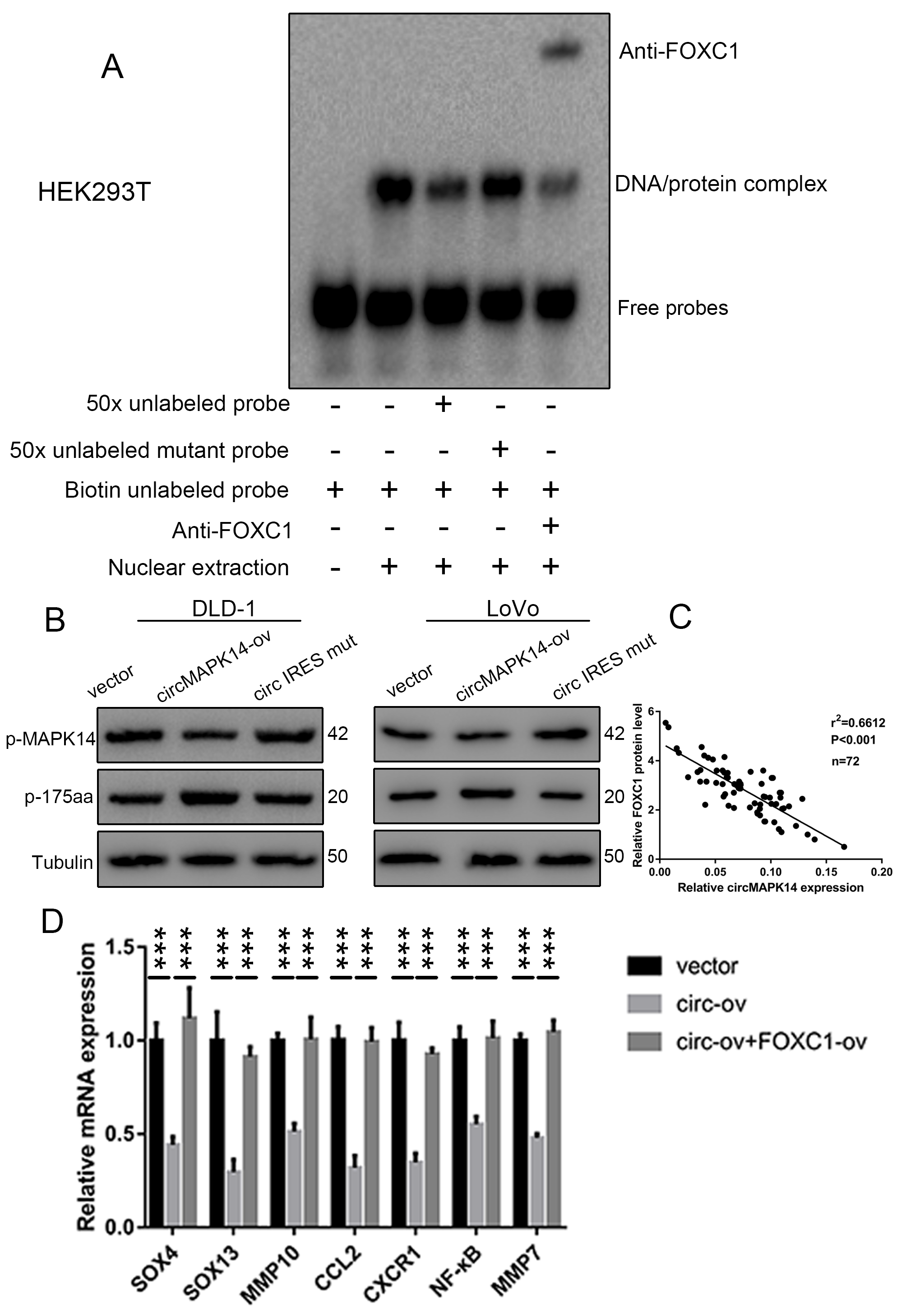

Supplement: Supplementary file 10 — SUPPORTING INFORMATION [file CTM2-11-e613-s001.tif]

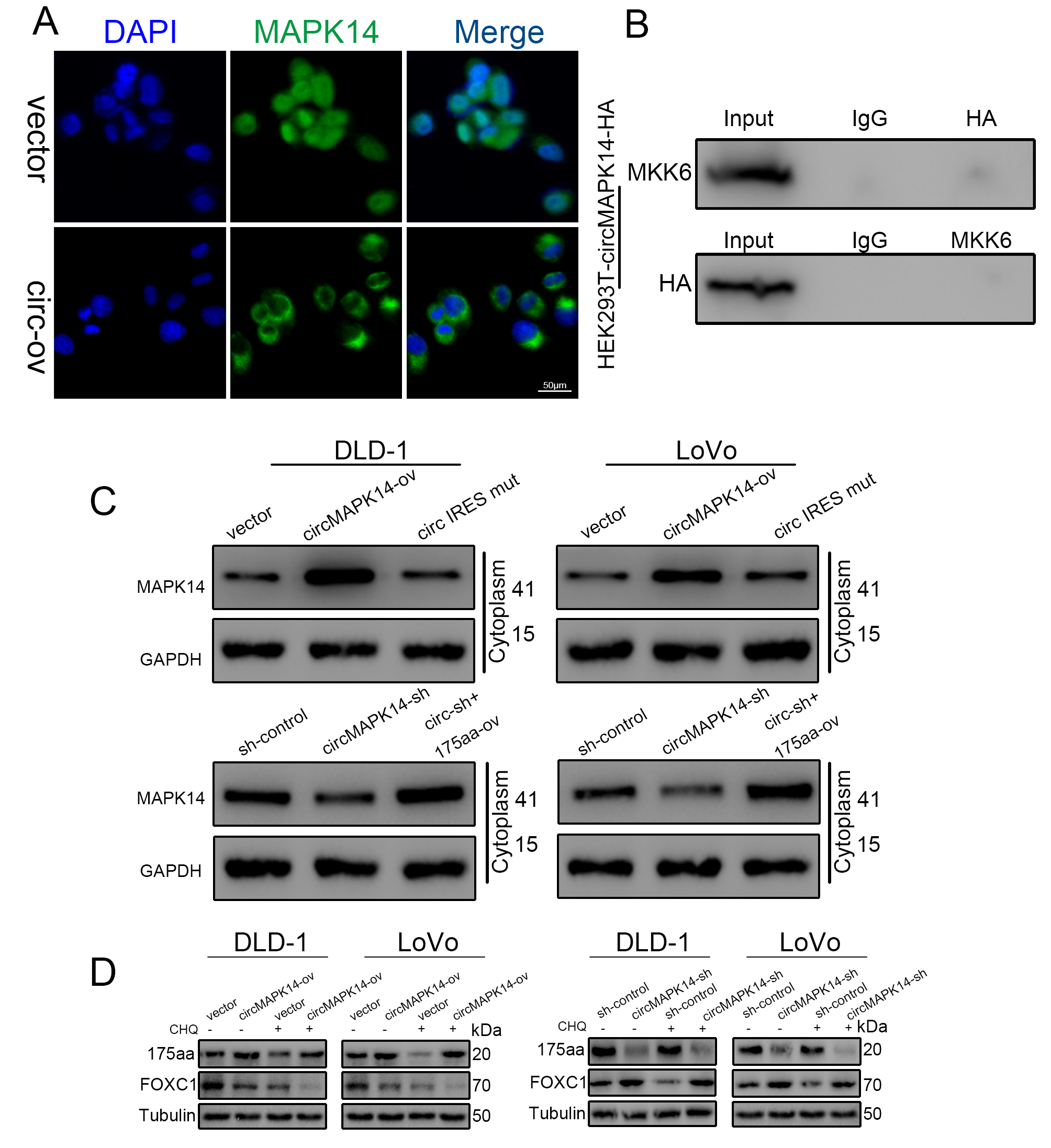

Supplement: Supplementary file 11 — SUPPORTING INFORMATION [file CTM2-11-e613-s010.tif]

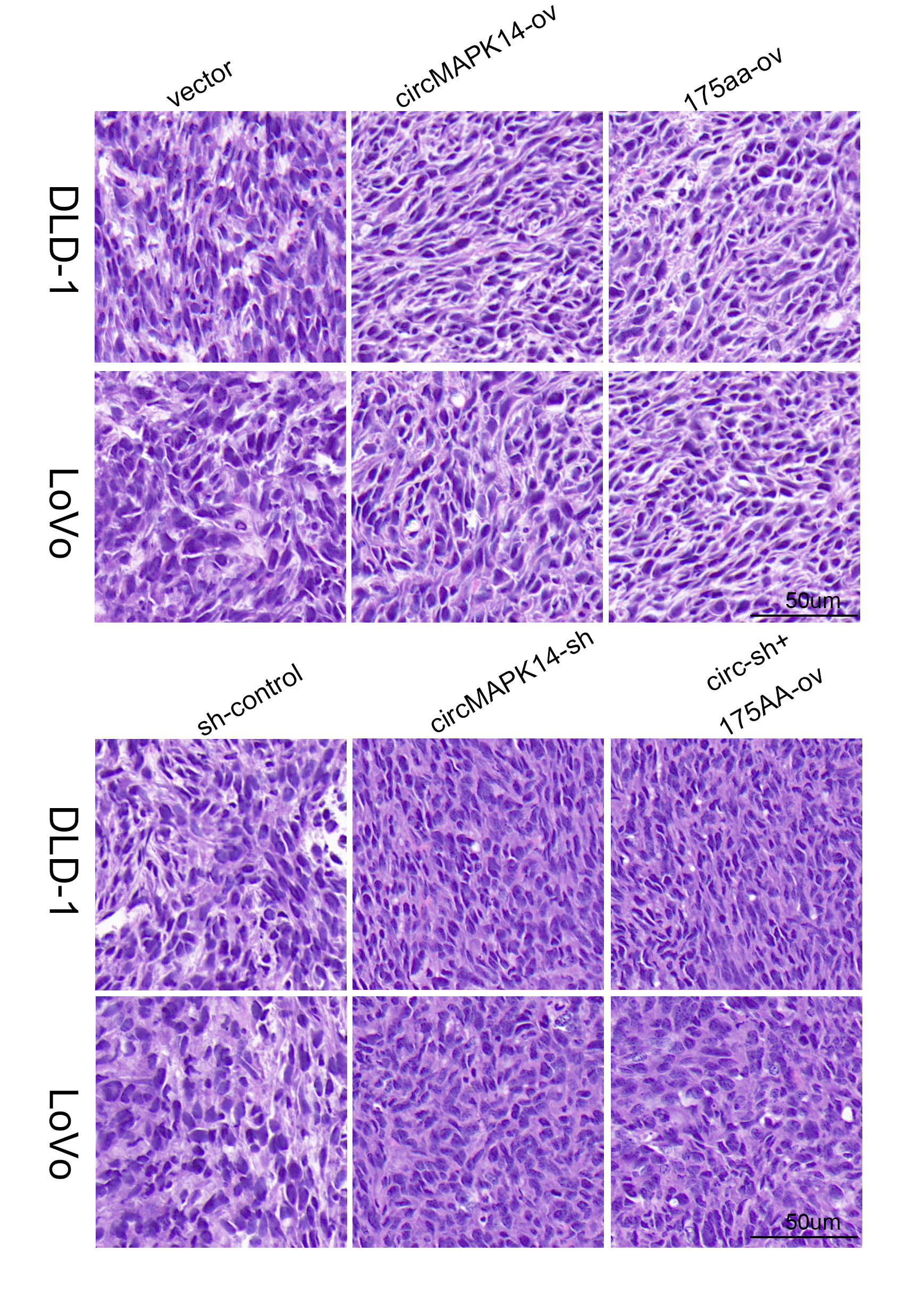

Supplement: Supplementary file 12 — SUPPORTING INFORMATION [file CTM2-11-e613-s005.tif]

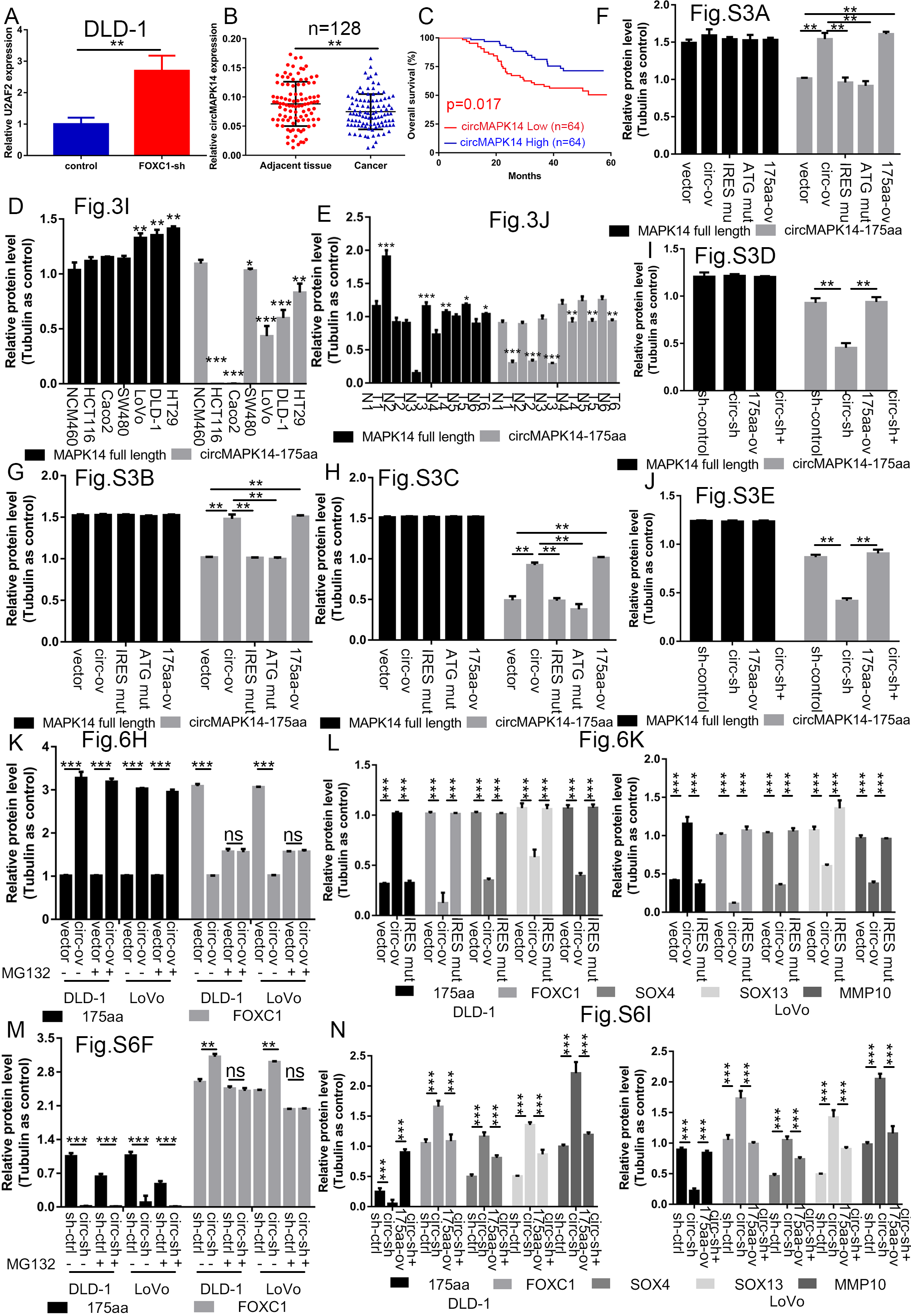

Supplement: Supplementary file 13 — SUPPORTING INFORMATION [file CTM2-11-e613-s014.tif]

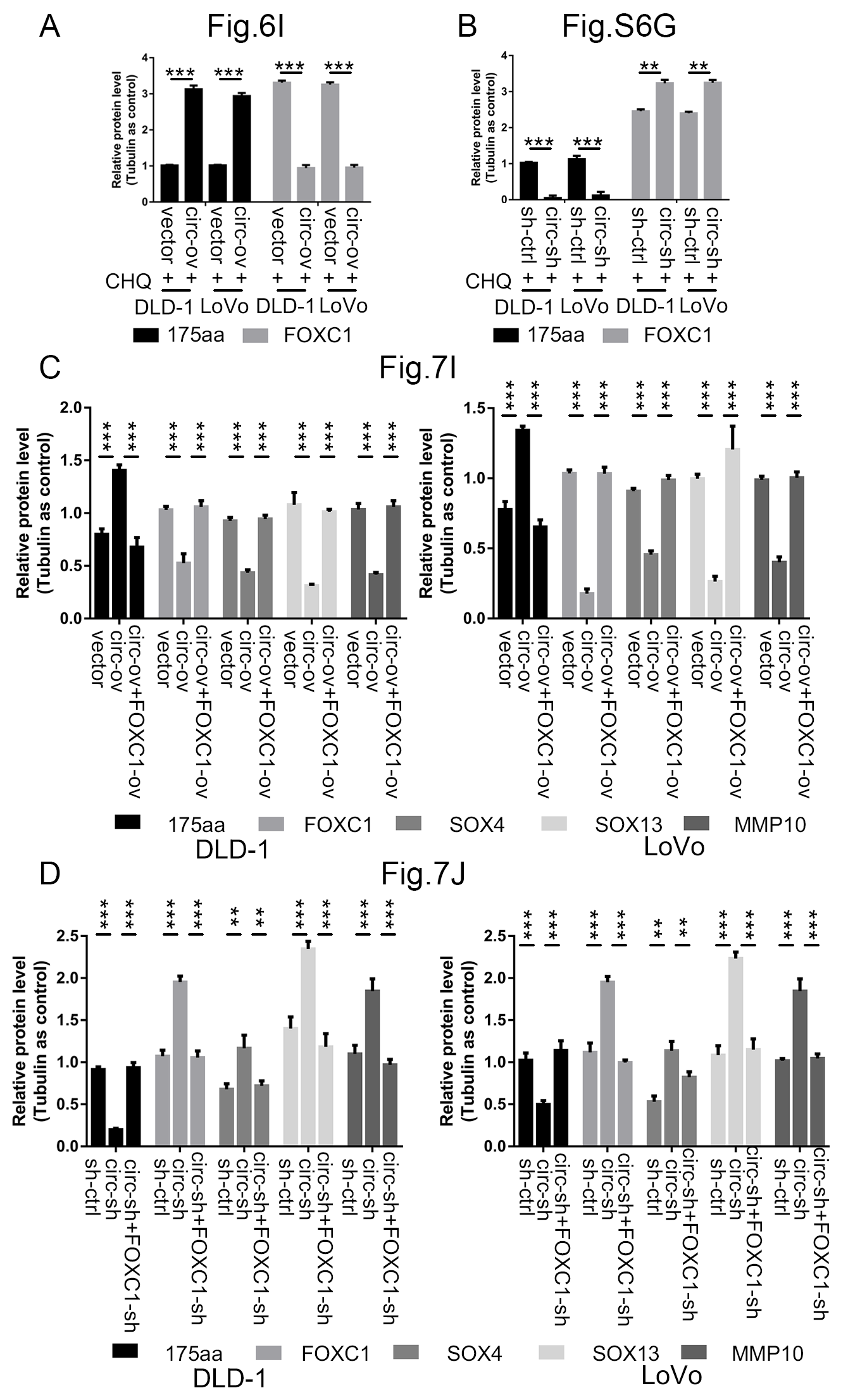

Supplement: Supplementary file 14 — SUPPORTING INFORMATION [file CTM2-11-e613-s007.tif]
