# Supplementary material for: A novel tumour suppressor protein encoded by circMAPK14 inhibits progression and metastasis of colorectal cancer by competitively binding to MKK6
Source: Clin Transl Med. 2021 Oct 14;11(10):e613. doi: 10.1002/ctm2.613 (PMC8516360; doi:10.1002/ctm2.613)
Supplement: Supplementary file 15 — SUPPORTING INFORMATION [file CTM2-11-e613-s004.docx]

| RT-PCR primers | Forward primer (5’ to 3’) | Reverse primer (5’ to 3’) |
| --- | --- | --- |
| circMAPK14  (divergent primers) | AGTCTTTGACTCAGATGCCGA | TGGTAGATAAGGAACTGAACATGG |
| circMAPK14  (convergent primers) | GATTTTGGACTGGCTCGGCA | GCCACGTAGCCTGTCATTTC |
| hsa_circ_15030 | TGTTGGACGTTTTTACACCTGC | TGATGGACTGAAATGGTCTGGA |
| hsa_circ_14100 | GTGGGATGCATAATGGCCGA | TCGCATGAATGATGGACTGAA |
| hsa_circ_27976 | TGGATGCATTACAACCAGACA | GTTCAGATCTGCCCCCATGA |
| MAPK14 | GCGGAGAGGTTCCATATTGGG | CTAAGGCTCCAGTGGTCGC |
| GAPDH | CCATGGGGAAGGTGAAGGTC | GACTCCACGACGTACTCAGC |
| Primer 1 | CAGGCCAAGTCTGGAGGATA | GCGAGATTTCCCTTACAACG |
| Primer 2 | TCGAGACCATCCTGGCTAAC | GGGTAGTGTCCACGCTTCTT |
| Primer 3 | TAAACTTGGCCTCAGCCTCT | CTCTGCCTCCGAGTTTTCTG |
