# Supplementary material for: A novel tumour suppressor protein encoded by circMAPK14 inhibits progression and metastasis of colorectal cancer by competitively binding to MKK6
Source: Clin Transl Med. 2021 Oct 14;11(10):e613. doi: 10.1002/ctm2.613 (PMC8516360; doi:10.1002/ctm2.613)
Supplement: Supplementary file 16 — SUPPORTING INFORMATION [file CTM2-11-e613-s012.docx]

**Supplementary Methods:**

**Apoptosis assay**

To conduct a cell apoptosis assay, cells were transfected under different conditions. Approximately 48 hours post-transfection, they were washed two times using PBS, and then treated with trypsin (without EDTA). Cells were reconstituted in a binding solution and then stained with 5 µl of Annexin V-FITC (Beyotime, China) reagent, followed by incubation in a dark place at room temperature (RT) for 15 minutes. Next, 1 µl of propidium iodide (PI, 50 µg/ml) (Beyotime, China) was added and gently mixed, followed by incubation in darkness at RT for 5 min, and submission for detection. Flow cytometry (FACScan, BD Biosciences) containing the Cell-Quest software was used to analyze the apoptotic cells.

**Electrophoresis mobility shift assay (EMSA)**

Gel electrophoresis mobility shift assay (EMSA) was carried out the instructions of the manufacturer (GS009, Beyotime Biotechnology, Shanghai, China). Concisely, the biotin-labeled probe was incubated in the 1× gel/DNA shift binding buffer containing 5 mM MgCl2, 50 mM KCl, 2.5% glycerol, and 10 mM EDTA with or without CmANR1 protein at 24 °C for 30 min. The unlabeled probe with specified concentrations (50×) was used for cold probe competition. EMSA probe:

GCCAGGGGGCAGTAACGCCAGATCATCTGTCTCCCGGAAGTAATGCCAGA

EMSA mutant probe:

GCCAGGGGGCATCGACGCCAGATCATCTGTCTCCCGGAATCGATGCCAGA

**Additional antibodies**

CCL2: ab214819 1:1000; CXCR1: ab124344 1:500; NFkB: ab32536 1:1000; MMP7 ab207299 1:1000.

The sequence of shRNA circMAPK14-sh#1:

| **ID** | **5’** | **stem** | **loop** | **stem** | **3’** |
| --- | --- | --- | --- | --- | --- |
| circRNA-RNAi-a | GATCCC | AATCCCCTGGGTATCTGGTGA | CTCGAG | TCACCAGATACCCAGGGGATT | TTTTTGGAT |
| circRNA-RNAi-b | AGCTATCCAAAAA | AATCCCCTGGGTATCTGGTGA | CTCGAG | TCACCAGATACCCAGGGGATT | GG |

The primers for the ORF construct:

| **ID** | **seq** |
| --- | --- |
| F | CGCAAATGGGCGGTAGGCGTG |
| R | TTATTAGGAAAGGACAGTGGG |

The primers for the IRES mut construct:

| **ID** | **seq** |
| --- | --- |
| F | CGCAAATGGGCGGTAGGCGTG |
| R | CAACTGATCAATATGGTCTG |
